# Mating proximity blinds threat perception

**DOI:** 10.1038/s41586-024-07890-3

**Published:** 2024-08-28

**Authors:** Laurie Cazalé-Debat, Lisa Scheunemann, Megan Day, Tania Fernandez-d.V. Alquicira, Anna Dimtsi, Youchong Zhang, Lauren A. Blackburn, Charles Ballardini, Katie Greenin-Whitehead, Eric Reynolds, Andrew C. Lin, David Owald, Carolina Rezaval

**Affiliations:** 1 School of Biosciences, University of Birmingham, Birmingham, UK; 2 Birmingham Centre for Neurogenetics, University of Birmingham, Birmingham, UK; 3 Freie Universität Berlin, Institute of Biology, Berlin, Germany; 4 Institut für Neurophysiologie and NeuroCure Cluster of Excellence, Charité — Universitätsmedizin Berlin, corporate member of Freie Universität Berlin and Humboldt–Universität zu Berlin, Berlin, Germany; 5 School of Biosciences, University of Sheffield, Sheffield, UK; 6 Neuroscience Institute, University of Sheffield, Sheffield, UK; 7 Present Address: Biosciences Institute, Newcastle University, Newcastle upon Tyne, UK; 8 Present Address: Centre for Neural Circuits and Behaviour, University of Oxford, Oxford, UK; 9 Present Address: School of Science and the Environment, University of Worcester, Worcester, UK

**Keywords:** Animal behaviour, Neural circuits, Sexual behaviour

## Abstract

Romantic engagement can bias sensory perception. This ‘love blindness’ reflects a common behavioural principle across organisms: favouring pursuit of a coveted reward over potential risks^1^. In the case of animal courtship, such sensory biases may support reproductive success but can also expose individuals to danger, such as predation^2,3^. However, how neural networks balance the trade-off between risk and reward is unknown. Here we discover a dopamine-governed filter mechanism in male *Drosophila* that reduces threat perception as courtship progresses. We show that during early courtship stages, threat-activated visual neurons inhibit central courtship nodes via specific serotonergic neurons. This serotonergic inhibition prompts flies to abort courtship when they see imminent danger. However, as flies advance in the courtship process, the dopaminergic filter system reduces visual threat responses, shifting the balance from survival to mating. By recording neural activity from males as they approach mating, we demonstrate that progress in courtship is registered as dopaminergic activity levels ramping up. This dopamine signalling inhibits the visual threat detection pathway via Dop2R receptors, allowing male flies to focus on courtship when they are close to copulation. Thus, dopamine signalling biases sensory perception based on perceived goal proximity, to prioritize between competing behaviours.

## Abstract (web summary)

A state-dependent dopamine filter system in the male *Drosophila* brain balances threat perception against the drive to mate.

## Main

Every day animals make decisions that require balancing opportunities and risks. This trade-off has been explored in humans^4,5^, rodents^6,7^ and invertebrates^8–14^. However, we still lack a detailed mechanistic understanding of how conflict is resolved in the brain, particularly when the dangers and benefits are crucial life choices.

One especially important trade-off is between survival and reproduction. Avoiding threats can be a life-saving decision, but excessive caution might result in missed mating opportunities. Recent work has revealed how sex drive and threat avoidance are independently signalled in the brain^15–21^, yet it remains unclear how these needs are prioritized when they are in conflict. How animals suppress courtship when it is better to run away, and how this is reversed when the rewards of courtship outweigh the risk of predation (for example, if mating is imminent) still remain unknown.

Dopamine is a key player in motivation, need and reward^22–24^. Beyond these functions, dopamine is thought to relay the value of sensory input and internal/behavioural states to decision-making centres, thus adapting behaviour^23,25,26^. Yet, how dopamine dynamically modulates sensory valence and influences decision-making during conflict remains poorly understood. This task could be mediated through sensory filter systems^27^, which block superfluous inputs and highlight relevant information to facilitate appropriate behaviours. Such filtering systems could thus serve as a means to shut down competing sensory inputs when animals are close to achieving a crucial goal. Here we describe a state-dependent filter system driven by dopamine that allows *Drosophila* males to filter out external threats and focus on courtship when they are close to mating.

## Visual threats block courtship via LC16

**Fig. 1:**
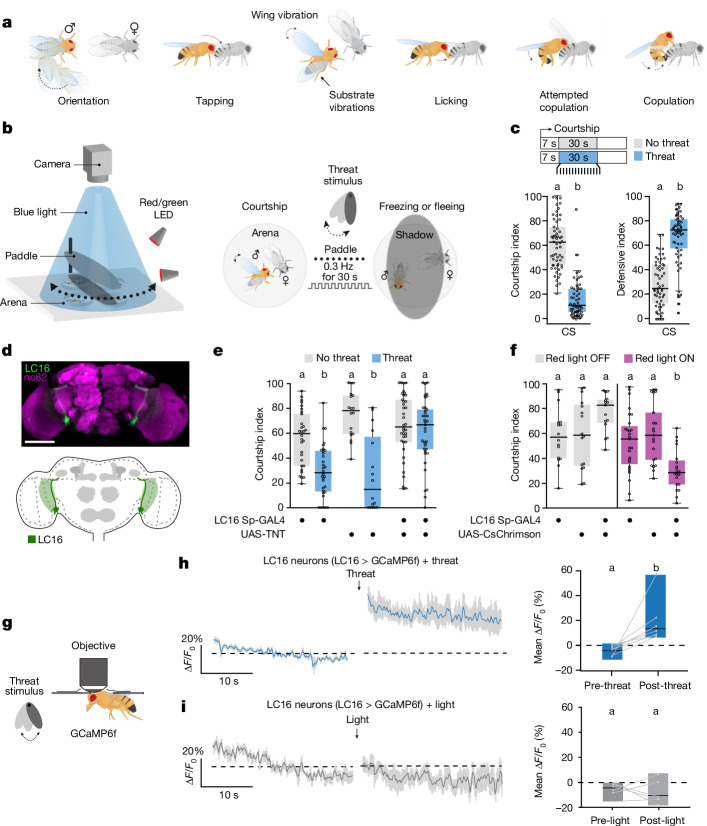
Courtship is disrupted by visual threats in male flies via LC16 neurons. **a**, Schematic of the courtship ritual. **b**, Schematic of the action selection paradigm. **c**, Courtship and defensive indices of wild-type males without (grey) or with (blue) the visual threat (*n* = 59 each). CS, Canton-S. **d**, LC16 split-GAL4 (Sp-GAL4) driving UAS-mCD8-GFP (green) in the adult brain; neuropil counterstaining is with anti-nC82 (magenta). Scale bar, 50 μm. **e**, LC16 > TNT flies fail to stop courting in response to the visual threat (*n* = 30, 18 and 38 (no threat); *n* = 32, 16 and 37 (threat)). **f**, LC16 > CsChrimson flies interrupt courtship upon artificial activation without threat (*n* = 17, 16 and 15 (red light OFF); *n* = 27, 21 and 20 (red light ON)). **g**, Schematic of live imaging with threat delivery. **h**,**i**, Left, ∆*F*/*F*_0_ (%) of the LC16 > GCaMP6f signal before and after presenting a threat (**h**) or a non-threat light (**i**). Mean ∆*F*/*F*_0_ (%) pre-stimulus and post-stimulus (*n* = 7 and 6) is also shown (right). The sample sizes represent biologically independent animals. The solid line and shaded area of live-imaging traces show mean ± s.e.m., respectively. Behavioural indexes are displayed as boxplots. The boxes delimit the lower (25th) and upper (75th) interquartile, and the horizontal line represents the median. Calcium imaging quantification plots are shown as minimum/maximum plots, and the median as a horizontal line. Each dot represents a single fly. Significant differences are indicated by different letters at the level of *P* < 0.05 (for example, a is different from b but not from ab). See Supplementary Table 1 for details on statistics.

*Drosophila* males engage in a series of stereotyped, progressive courtship steps to achieve copulation^17^ (Fig. 1a and Supplementary Videos 1 and 2). If the female is receptive, the male typically exhibits strong courtship motivation^16,19,28^, persisting until copulation is achieved. Yet, what happens when the urge to court is fraught with risk?

**Extended Data Fig. 1:**
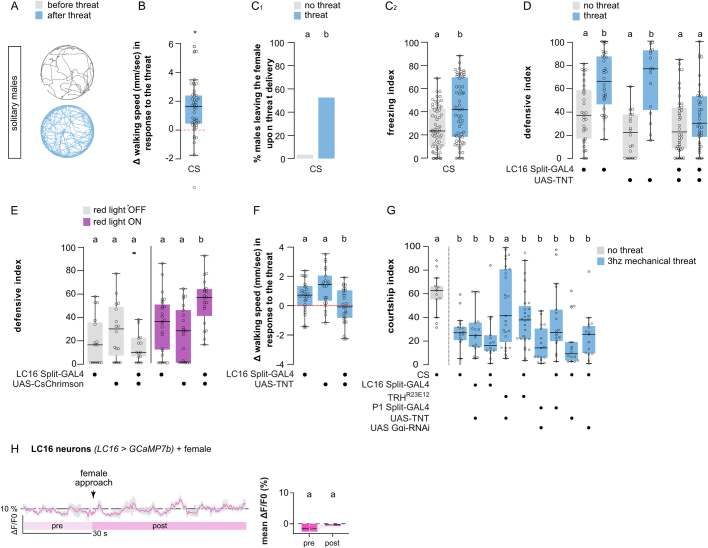
LC16 neurons detect visual threats and trigger defensive behaviours in male *Drosophila.* **A**, Locomotion traces of solitary wild-type males before and after threat (n = 8). **B**, Change in walking speed of wild-type males before and after threat (n = 39). **C**, Percentage of wild-type males leaving the female **(C**_**1**_**)** and freezing index **(C**_**2**_**)** without (grey bar, n = 59) or with the threat (blue bars, n = 59). **D**, Defensive index of LC16 > TNT males and controls without (grey bars, n = 30,18,38) or with the threat (blue bars, n = 32,16,37). **E**, Defensive index after artificial activation of LC16>CsChrimson without the threat (n^red light OFF^ = 17,16,15; n^red light ON^ = 27,21,20) and controls. **F**, Change in walking speed of LC16 > TNT males and controls before and after threat (n = 28,23,28). **G**, Courtship indexes of wild type and transgenic males exposed to a mechanical threat (n = 13,17,17,14,14,27,28,17,15,18). **H**, Left: ∆F/F0% of LC16 > GCaMP7b signal pre and post exposure to a female fly. Right: mean ∆F/F0% comparing pre and post time windows (n = 5). The sample sizes represent biologically independent animals. Solid line and shaded area of live-imaging traces show mean ± s.e.m respectively. Boxes delimit the lower (25th) and upper interquartile (75th), and the horizontal line represents the median. Calcium imaging quantification plots are shown as min/max plots and median as horizontal line. Each dot represents a single fly. Significant differences are indicated by different letters at the level of p < 0.05 (e.g: a is different from b but not from ab). See Supplementary Table 1 for details on statistics.

To dissect the neural circuitry that prioritizes between sex and survival, we established a sex–danger conflict assay, in which courting *Drosophila* males were presented with a visual threat: a predator-like moving shadow^29^ (Fig. 1b). Indeed, in the absence of females, the threat caused males to show defensive responses such as running away and freezing^29^ (Extended Data Fig. 1a,b). To eliminate confounding effects of female behaviour, we used immobile virgin females. As expected, in the absence of the threat, wild-type males vigorously courted the females and showed low defensive behaviours (Fig. 1c, Extended Data Fig. 1c and Supplementary Video 1). However, upon threat presentation, males immediately halted courtship and engaged in defensive responses (Fig. 1c, Extended Data Fig. 1c and Supplementary Videos 3 and 4).

We next asked which neurons detect the visual threat. Lobular columnar (LC) visual projection neurons connect early visual processing with central brain areas and respond to conspecifics and motion cues^15,20,21,30^. We therefore hypothesized that LC neurons might detect and convey visual threats to higher brain centres to inhibit courtship. Indeed, we found that LC16 neurons^20,31^ were required to prioritize defensive responses over courtship (Fig. 1d,e and Extended Data Fig. 1d). When LC16 neurons were silenced by expression of tetanus toxin light chain (TNT), males courted the female despite the threat (Fig. 1e and Extended Data Fig. 1d). Conversely, when LC16 neurons were optogenetically activated using CsChrimson in the absence of the threat, males stopped courting, mimicking the effect of the visual threat (Fig. 1f and Extended Data Fig. 1e). Blocking LC16 synaptic output disrupted visual threat responses in solitary males (Extended Data Fig. 1f) but did not alter responses to mechanical threats in courting males (Extended Data Fig. 1g). Males with silenced LC16 neurons showed normal courtship behaviours in the absence of threats (Fig. 1e, grey plot). Therefore, LC16 neurons suppress courtship in response to visual threats by modulating courtship-related circuits, rather than directly controlling courtship.

LC16 neurons are sensitive to both looming cues and moving bars^20,32^. To verify that LC16 neurons can also perceive the moving shadow stimulus, we performed in vivo two-photon calcium imaging (Fig. 1g). Visual threats induced substantial calcium influx in LC16 neurons, whereas non-threat stimuli such as light-only controls or female sensory cues did not (Fig. 1h,i, Extended Data Fig. 1h and Supplementary Table 2). Together, our findings show that LC16 neurons detect visual threats and prompt the flies to cease courtship and engage in defensive actions.

## Prioritizing survival requires 5-HT

**Extended Data Fig. 2:**
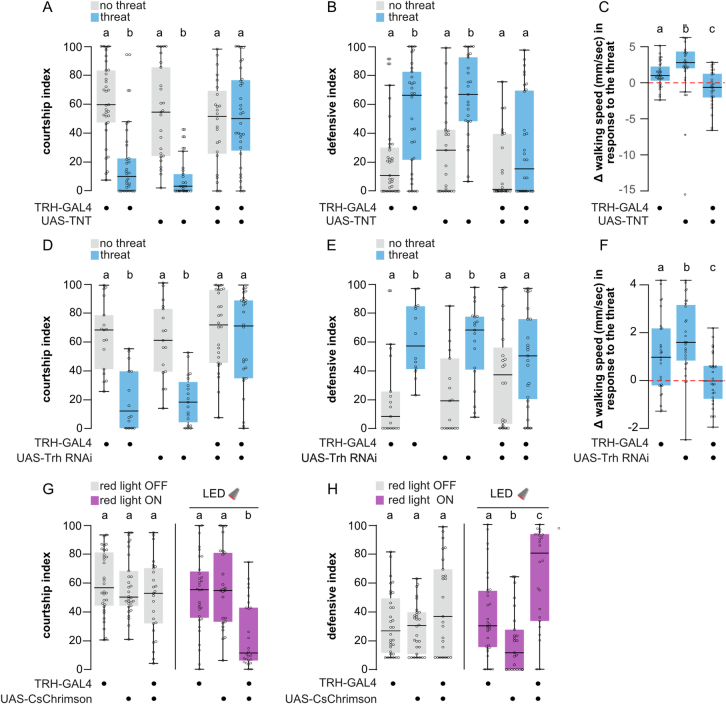
5-HT neurons are required to prioritize survival over courtship. **A**,**B**, Courtship and defensive indexes TRH > TNT males without (grey plots, n = 33,25,24) or with the threat (blue plots, n = 30,25,32) and controls. **C**, Change in walking speed TRH > TNT males and controls before and after threat. (n = 32,30,28). **D**,**E**, Courtship and defensive indices TRH > TRH-RNAi males and controls without (grey plots, n = 17,17,26) or with a threat (blue plots, n = 14,19,26). **F**, Change in walking speed of TRH > TRH-RNAi males and controls before and after threat (n = 27,28,28). **G**,**H**, Courtship and defensive indexes of TRH>CsChrimson upon artificial activation without the threat (n^red light OFF^ = 32,30,25; n^red light ON^ = 31,25,26) and controls. See legend to Extended Data Fig. 1 for details on graphics and statistics.

Previous research in fish and mammals has shown that serotonin (5-HT) signalling is increased by stress and modulates predator-associated fight-or-flight responses^33,34^. Therefore, we postulated that inhibition of courtship could be driven by 5-HT signalling. To test this hypothesis, we either silenced all 5-HT neurons or blocked 5-HT synthesis altogether using RNA interference (RNAi) against tryptophan hydroxylase (TRH), an enzyme required for 5-HT synthesis. In both cases, the threat did not increase walking speed in solitary males or stop courtship in males paired with females (Extended Data Fig. 2a–f). By contrast, optogenetically activating 5-HT neurons stopped courtship in the absence of the threat (Extended Data Fig. 2g,h). These experiments indicate that 5-HT neurons are important for prioritizing escape over courtship in response to visual threats.

## 5-HT inhibits central courtship nodes

**Fig. 2:**
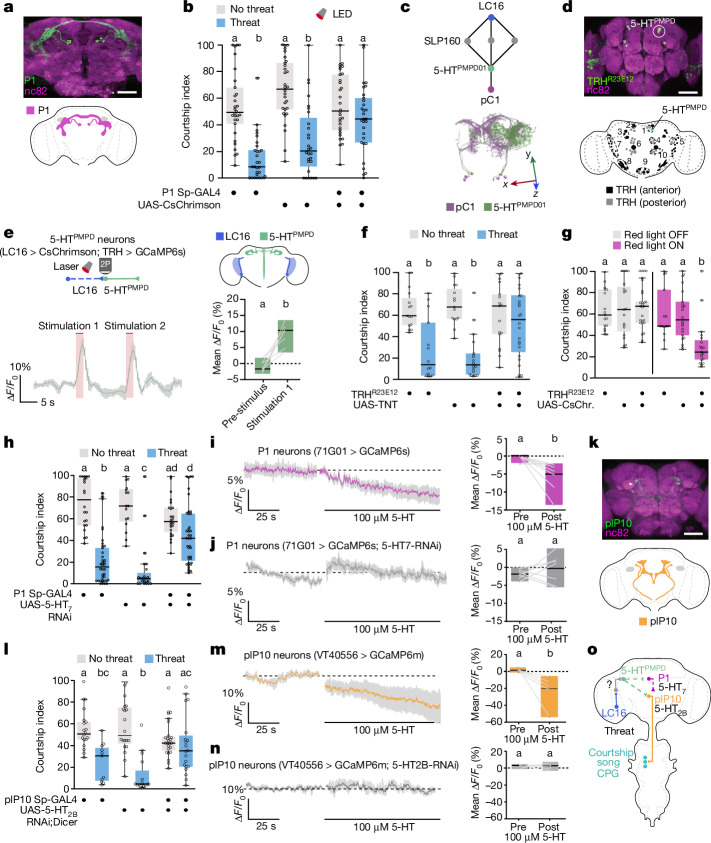
Visually driven 5-HT signalling inhibits P1 and plP10 courtship-promoting neurons. **a**, P1 Sp-GAL4-driving UAS-mCD8-GFP (green) in the adult brain. Anti-nC82 is in magenta. Scale bar, 50 μm. **b**, P1 > CsChrimson males continue courtship upon artificial activation despite the threat (*n* = 30 each (no threat); *n* = 30, 28 and 30 (threat)). **c**, Predicted path connecting LC16 to pC1a through 5-HT^PMPD01^ (top), and electron microscope reconstructions of female pC1 and 5-HT^PMPD01^ neurons (bottom). See Supplementary Information Fig. 2c for axis details. **d**, TRH^R23E12^ Sp-GAL4 labels PMPD neurons (top), and 5-HT clusters in the adult brain (bottom). 1 denotes the posterior medial dorsal protocerebrum (PMPD) cluster. See Supplementary Information Fig. 2d for the nomenclature for clusters. **e**, ∆*F*/*F*_0_ (%) of the 5-HT^PMPD^ > GCaMP6s signal after artificial activation of LC16 neurons (left), and the mean ∆*F*/*F*_0_ (%) at baseline versus first stimulation (*n* = 7). **f**, TRH^R23E12^ > TNT males fail to stop courting in response to the threat (*n* = 15, 15 and 17 (no threat); *n* = 14, 19 and 24 (threat)). **g**, TRH^R23E12^ > CsChrimson (CsChr.) males stop courtship upon artificial activation without threat (*n* = 17, 19 and 25 (red light OFF); *n* = 14, 22 and 19 (red light ON)). **h**, P1 > 5-HT_7_ RNAi flies respond less to the threat (*n* = 22, 16 and 24 (no threat); *n* = 39, 24 and 39 (threat)). **i**,**j**, ∆*F*/*F*_0_ (%) of the P1 > GCaMP6s signal pre-application and post-application of 100 µM 5-HT without (**i**) or with (**j**) the 5-HT_7_ receptor knocked down in P1 neurons. The mean ∆*F*/*F*_0_ (%) comparing the before and after application of 5-HT time windows (*n* = 7 and 7) is also shown (right). **k**, plP10 Sp-GAL4 driving UAS-mCD8-GFP (green) in the adult brain. Anti-nC82 is in magenta. Scale bar, 50 μm. **l**, plP10 > 5-HT_2B_-RNAi;Dicer males respond less to the threat (*n* = 18, 20 and 24 (no threat); *n* = 11, 15 and 20 (threat)). **m**,**n**, ∆*F*/*F*_0_ (%) of the P1 > GCaMP6m signal pre-application and post-application of 100 µM 5-HT without (**m**) or with (**n**) the 5-HT_2B_ receptor knocked down in p1P10 neurons. The mean ∆*F*/*F*_0_ (%) comparing the before and after application of 5-HT time windows (*n* = 6 and 6) is also shown (right). **o**, Network model. The dashed lines indicate non-functionally established connections. CPG, central pattern generator. Refer to the legend of Fig. 1 for details on graphics and statistics.

**Extended Data Fig. 3:**
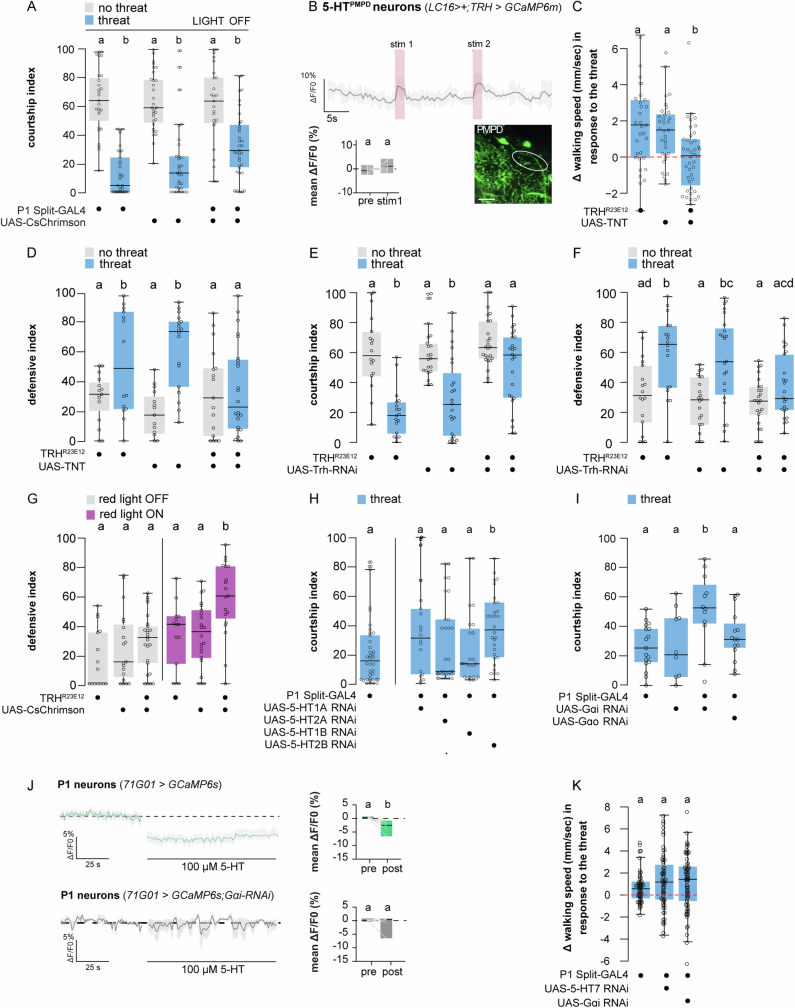
Visually-driven 5-HT^PMPD^ neurons inhibit the P1 central courtship node via 5-HT7- Gα_i_ signalling. **A**, Courtship index of P1>CsChrimson males without (grey bars, n = 27,31,25) or with (blue bars, n = 34,31,35) the threat (red light OFF). **B**, ∆F/F0% of TRH^PMPD^ > GCaMP6m signal upon laser stimulation. Left: mean ∆F/F0% signal comparing baseline to stimulation (n = 9). Right: focal plane showing ROI during imaging (scale bar: 15 µm) refer to Fig. 2e. **C**, Change in walking speed of TRH^R23E12^ > TNT males and controls before and after threat (n = 31,33,40). **D**, Defensive index of TRH^R23E12^ > TNT males and controls without (grey bars, n = 15,15,17) or with the threat (blue bars, n = 14,19,24). **E**,**F**, Courtship and defensive indexes of TRH > TRH-RNAi males and controls without (grey plots, n = 18,21,25) or with threat (blue plots, n = 18,23,25). **G**, Defensive index of TRH^R23E12^>CsChrimson males upon artificial activation without threat (n^red light OFF^ = 17,19,25; n^red light ON^ = 14, 22, 19) and controls. **H**, Behavioural effects of knocking-down 5-HT receptors in P1 neurons using RNAi, in control (n = 38) and experimental males (n = 18,15,12,29). **I**, Behavioural effects of knocking-down Gα_i_ and Gα_o_ proteins in P1 neurons using RNAi (n = 9,12,15), and controls (n = 17). **J**, Upper: ∆F/F0% of P1 > GCaMP6s signal pre and post application of 100 µM 5-HT and with simultaneous knock-down of Gα_i_ protein **(lower)**. Right: mean ∆F/F0% comparing the pre and post time windows (n = 6,7). **K**, Change in walking speed of P1 > 5-HT7-RNAi and P1>Gα_i_-RNAi males before and after threat (n = 58,64,70). See legend to Extended Data Fig. 1 for details on graphics and statistics.

In our search for the neurons involved in the courtship–escape choice, we examined the P1 cluster, a central mating regulation hub that initiates courtship in response to female sensory cues and internal states^15,17,19,28,35,36^ (Fig. 2a). Given that P1 neurons integrate other competing drives^9,36–38^, we hypothesized that they may also regulate the courtship–escape choice. Indeed, optogenetically activating P1 neurons in males during the exposure to the visual threat caused them to continue to court, overriding the threat response (Fig. 2b and Extended Data Fig. 3a). This result suggests that visual threats might block courtship by inhibiting P1 neurons.

Using the female *Drosophila* connectome^39^, we identified a 5-HT neuron (5-HT^PMPD01^) in the posterior medial dorsal (PMPD) cluster (Fig. 2c,d) that receives input from LC16 neurons through an intermediate neuron and that in turn synapses onto pC1, the female equivalent of P1 (Fig. 2c). Although the same connection is not guaranteed to exist on male P1 neurons, thanks to the sexual dimorphism of pC1/P1, these connectome data made 5-HT^PMPD^ neurons attractive candidates for carrying threat signals from LC16 to P1.

To assess whether LC16 and 5-HT^PMPD^ neurons are functionally connected, we optogenetically activated LC16 and monitored 5-HT^PMPD^ responses by GCaMP6s calcium imaging (Fig. 2e). LC16 stimulation reliably increased the GCaMP6s signal in 5-HT^PMPD^ neurons (Fig. 2e), whereas light stimulation in flies lacking CsChrimson had a slight but not significant effect (Extended Data Fig. 3b and Supplementary Table 2), placing the 5-HT^PMPD^ cluster downstream of threat detecting neurons. Furthermore, threat exposure triggered significant calcium influx in 5-HT^PMPD^ neurons (Fig. 5k), similar to the threat response of LC16 neurons.

To test the role of 5-HT^PMPD^ neurons in threat-induced suppression of courtship, we used a split-GAL4 (TRH^R23E12^) targeting the 5-HT^PMPD^ cluster and a subset of 5-HT^LP^ neurons^40^ (Fig. 2d). Blocking TRH^R23E12^ synaptic output prevented solitary males from escaping threats (Extended Data Fig. 3c), implicating them in defensive responses. We further found that silencing TRH^R23E12^ neurons by either expressing TNT or knocking down the gene encoding the TRH enzyme led to persistent courtship activity in the presence of either a visual threat (Fig. 2f and Extended Data Fig. 3d–f) or a mechanical threat (Extended Data Fig. 1g). By contrast, optogenetic activation of TRH^R23E12^ in the absence of the threat caused male flies to terminate courtship and exhibit defensive behaviours (Fig. 2g and Extended Data Fig. 3g). Together, these data suggest that TRH^R23E12^ neurons may integrate threats of different modalities to act as general effectors of threat responses.

We next sought to identify the 5-HT receptor (or receptors) involved in inhibiting courtship. *Drosophila* have five highly conserved 5-HT G protein-coupled receptors (GPCRs)^41^. We individually downregulated each 5-HT receptor in P1 neurons using RNAi. Knocking down 5-HT_1A_, 5-HT_2A_ or 5-HT_1B_ did not significantly affect threat responses (Extended Data Fig. 3h). However, flies deficient in either 5-HT_7_ or 5-HT_2B_ receptors in P1 responded less to the threat and showed higher courtship levels than controls (Fig. 2h and Extended Data Fig. 3h). Given that knocking down 5-HT_7_ gave the strongest phenotype, we focused our analysis on this receptor. In live-imaging experiments, applying 5-HT to the brain decreased GCaMP6s fluorescence in P1 neurons (Fig. 2i), an inhibitory effect that was abolished by decreasing 5-HT_7_ expression in P1 (Fig. 2j and Supplementary Table 2). These experiments suggest that 5-HT suppresses courtship by inhibiting P1 cells via 5-HT_7_ (note that we do not exclude a role for 5-HT_2B_).

Although *Drosophila* 5-HT_7_ can act as an excitatory receptor by increasing intracellular cAMP, the same GPCR can be excitatory or inhibitory depending on the associated G protein and the cell type^41–43^. To investigate the mechanism by which 5-HT_7_ triggers inhibition, we downregulated different G proteins and evaluated behavioural responses. Knocking down the inhibitory Gα_i_ protein in P1 neurons prevented males from prioritizing defensive responses over courtship (Extended Data Fig. 3i) and abolished the inhibitory effect of 5-HT on P1 calcium activity (Extended Data Fig. 3j and Supplementary Table 2). Knocking down either 5-HT_7_ or Gα_i_ in P1 neurons did not affect the response of solitary males to the threat (Extended Data Fig. 3k). These findings collectively suggest that, in response to visual threats, 5-HT inhibits P1 via 5-HT_7_–Gα_i_ signalling, thereby suppressing courtship.

**Extended Data Fig. 4:**
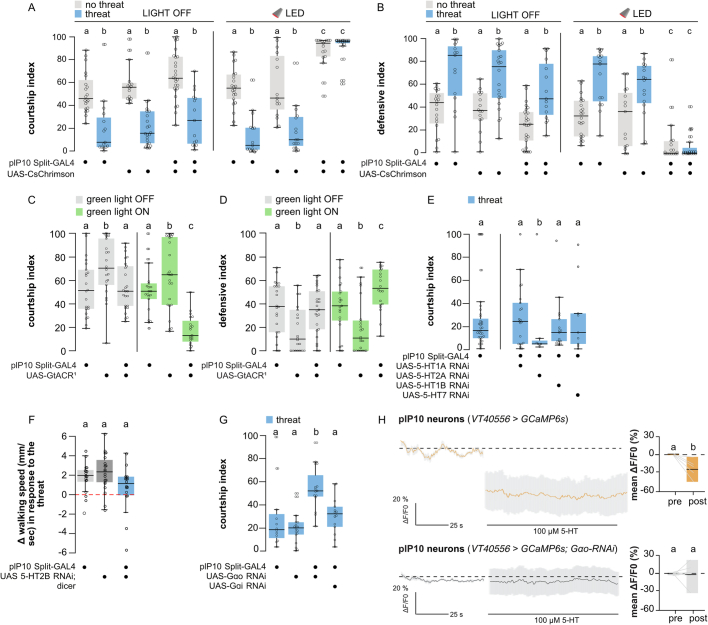
5-HT neurons inhibit plP10 courtship neurons via 5-HT2B-Gα_o_ signalling. **A**,**B**, Courtship **(A)** and defensive **(B)** indices without red light (left panel) or after artificial activation of plP10>CsChrimson (right panel) without (grey bars, n^light OFF^ = 20,16,26; n^light ON^ = 22,15.17) or with (blue bars, n^light OFF^ = 15,22,15; n^light ON^ = 15,16,20) the threat. **C**,**D**, Courtship **(C)** and defensive **(D)** indices of plP10 > GtACR^1^ males upon artificial inhibition without the threat (n^green light OFF^ = 22,22,25; n^green light ON^ = 21,24,20) and controls. **E**, Behavioural effects of knocking-down 5-HT receptors in plP10 neurons using RNAi, in control (n = 35) and experimental males (n = 21,7,17,9). **F**, Change in walking speed of plP10 > 5-HT2B-RNAi males and controls before and after threat (n = 18,20,18). **G**, Behavioural effects of knocking down Gα_i_ and Gα_o_ proteins in plP10 neurons using RNAi and controls (n = 12,14,15,18). **H**, Upper: ∆F/F0% of plP10 > GCaMP6s signal pre and post application of 100 µM 5-HT and with simultaneous knock-down of Gαo protein **(lower)**. Left: mean ∆F/F0% comparing the pre and post time windows (n = 5,7) See legend to Extended Data Fig. 1 for details on graphics and statistics.

Given that inhibiting P1 suppresses courtship only transiently^16^, we reasoned that other neurons are involved in sustaining the threat-induced inhibition of courtship. One potential candidate is plP10 descending neurons, which target the wing motor region in the ventral nerve cord and are crucial for courtship song^44^ (Fig. 2k). Indeed, optogenetic activation of plP10 resulted in high, sustained courtship levels throughout threat delivery (Extended Data Fig. 4a,b), whereas optogenetic inhibition of plP10 via GtACR1 in the absence of the threat robustly suppressed courtship (Extended Data Fig. 4c,d).

We next asked whether — as with P1 neurons — plP10-mediated courtship inhibition is serotonergic. Knocking down the 5-HT_2B_ receptor in plP10 neurons by RNAi resulted in males continuing courtship despite the threat (Fig. 2l and Extended Data Fig. 4e), although it did not affect defensive behaviours in solitary males (Extended Data Fig. 4f). Knocking down other 5-HT receptors did not significantly affect the behavioural choice (Extended Data Fig. 4e). In calcium imaging experiments, 5-HT application significantly decreased the activity of plP10 neurons (Fig. 2m), an inhibitory effect that was abolished by reducing 5-HT_2B_ expression in plP10 neurons (Fig. 2n and Supplementary Table 2). Together, these results show that 5-HT inhibits plP10 neurons via 5-HT_2B_.

Like 5-HT_7_, 5-HT_2B_ is not considered to be an inhibitory receptor^41^. To elucidate how 5-HT_2B_ can inhibit plP10, we downregulated different G proteins. Depleting the inhibitory Gα_o_ protein — but not Gα_i_ — from plP10 neurons prevented males from prioritizing escape responses over courtship (Extended Data Fig. 4g) and abolished the inhibitory effect of 5-HT on plP10 calcium activity (Extended Data Fig. 4h and Supplementary Table 2). Together, these findings suggest that 5-HT_2B_ inhibits plP10 via Gα_o_.

Although direct evidence for connections between 5-HT^PMPD^, P1 and plP10 neurons awaits confirmation, our findings suggest a model in which, upon threat detection, LC16 neurons activate 5-HT^PMPD^ neurons via an intermediate neuron. The threat-driven release of 5-HT inhibits P1 and plP10 neurons, allowing flies to prioritize survival over sex (Fig. 2o). Of note, threat-driven inhibition of courtship was not fully prevented by blocking serotonergic signalling to either P1 or plP10 individually (Fig. 2h,l), indicating that either serotonergic pathway acting alone can partially suppress courtship upon threat detection.

## Males ignore threats late in courtship

**Fig. 3:**
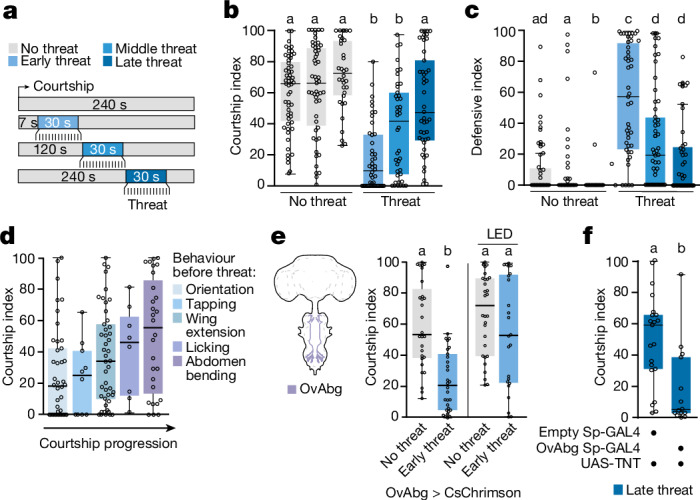
Flies engaged in later courtship steps show reduced threat responses. **a**, Behavioural protocol. The threat is delivered after 7 s (early), 120 s (middle) or 240 s (late) of sustained courtship. The no threat controls have been tested at the same timepoints. **b**,**c**, Courtship (**b**) and defensive (**c**) indices of wild-type males with no threat (grey; *n* = 56, 55 and 48) or with an early (30 s), middle (120 s) or late (240 s) threat (blue; *n* = 47, 50 and 52). **d**, Courtship index of wild-type males from panels **a**,**b** displaying a given behaviour before the threat, irrespective of the stage of threat delivery (*n* = 42, 10, 46, 8 and 28). **e**, OvAbg > CsChrimson males continue courtship upon artificial activation despite the threat (*n* = 28 and 30 (no threat); *n* = 30 and 24 (threat)). **f**, OvAbg > TNT males respond to a late threat (240 s; *n* = 21 and 14). Refer to the legend of Fig. 1 for details on graphics and statistics.

**Extended Data Fig. 5:**
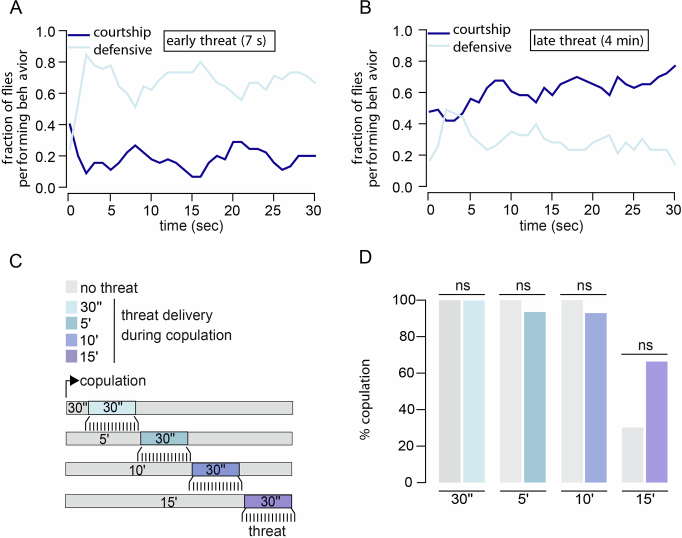
Late courtship stages and copulation decrease threat responses. **A**,**B**, Fraction of flies exhibiting courtship or defensive behaviour in response to an early **(A)** or late **(B)** threat, delivered after 7 s or 4 min of sustained courtship (n^early^ = 47, n^late^ = 52). **C**, Behavioural protocol: the threat is delivered at either 30 s, 5, 10 or 15 min after copulation initiation. Controls for copulation have been observed at the same time point in absence of the threat. (Note that sperm transfer takes ~10 min^10^.) **D**, Percentage of flies engaged in copulation 30 s, 5, 10 or 15 min after mating initiation in absence (n = 10,10,10,10) or presence (n = 16,24,21,18) of the threat. See legend to Extended Data Fig. 1 for details on graphics and statistics.

Our findings show that male flies abort courtship in response to threats presented directly after courtship onset. We next asked whether this is also the case in advanced stages, when they have already invested in courtship and are probably closer to achieving mating. Indeed, previous research has shown that the value of sensory information can be influenced by ongoing behaviour and the internal state^15,22,23,25,26^. Therefore, to test whether male flies respond differently to the visual threat at advanced stages, we delivered the visual threat at progressive courtship stages (Fig. 3a). As males advanced further in the courtship process, the threat became less effective at making them stop courting and show defensive behaviours (Fig. 3b,c and Extended Data Fig. 5a,b). Moreover, copulating male flies completely ignored visual threats, even after sperm transfer (Extended Data Fig. 5c,d), in contrast to heat-shock threats, which do terminate copulation after sperm transfer^10^. These results collectively suggest that as courtship progresses, males become increasingly unresponsive to visual threats.

Female flies signal acceptance and readiness to copulate by ceasing rejection behaviours and slowing down, allowing the male to bend its abdomen and mount them. Thus, abdomen bending may indicate proximity to expected copulation^17^. We asked whether the late-courtship reduction in threat response was linked to the execution of advanced courtship steps. Indeed, males engaged in abdomen bending before threat presentation were less likely to interrupt courtship, regardless of when the threat was delivered (Fig. 3d and Supplementary Video 5). In line with this, optogenetically inducing abdomen bending by activating a small subset of abdominal ganglion neurons (OvAbg)^45^ also diminished threat responses (Fig. 3e). Crucially, silencing OvAbg neurons made flies reduce courtship in response to the threat even during late courtship stages, indicating that OvAbg activity is required for late-courtship males to ignore the threat (Fig. 3f).

## Dopamine ramps up during courtship

**Fig. 4:**
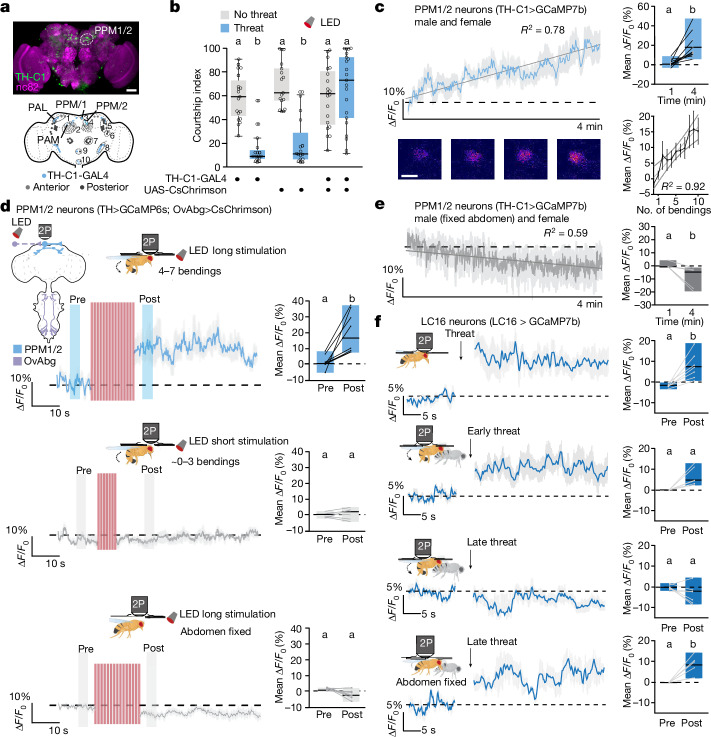
Ramping dopamine release reflects courtship progress. **a**, TH-C1-GAL4 driving UAS-mCD8-GFP (green) in the adult brain (top). Anti-nC82 is in magenta. Scale bar, 50 μm. The TH-C1-GAL4 expression pattern (blue) is also shown (bottom). See Supplementary Information Fig. 4a for the nomenclature of clusters. **b**, TH-C1 > CsChrimson males continue courtship upon artificial activation despite the threat (*n* = 19, 17 and 20 (no threat); *n* = 16, 16 and 21 (threat)). **c**, ∆*F*/*F*_0_ (%) of the TH-C1^PPM1/2^ > GCaMP7b signal of males exposed to females (top left); comparing mean ∆*F*/*F*_0_ (%) during the first minute (0–20 s) and at 4 min (220–240 s) (*n* = 10; top right); and a representative fluorescence heatmap of PPM1/2 neurons over time (bottom left). Scale bar, 2 µm. A correlation of calcium activity and the number of abdominal bendings is also shown (bottom right). **d**, ∆*F*/*F*_0_ (%) of the TH-C1^PPM1/2^ > GCaMP6s signal increases after long (top left) but not short (middle left) optogenetic stimulation of OvAbg > CsChrimson, and not when the abdomen is fixed (bottom left). The mean ∆*F*/*F*_0_ (%) during pre-stimulation and post-stimulation (*n* = 8) is also shown (right). See Extended Data Fig. 6m for details on the protocols. **e**, ∆*F*/*F*_0_ (%) of the TH-C1^PPM1/2^ > GCaMP7b signal of males with the abdomen fixed paired with a female (left), and comparing the mean ∆*F*/*F*_0_ (%) during the first minute and at 4 min (*n* = 6; right). **f**, ∆*F*/*F*_0_ (%) of the LC16 > GCaMP7b signal pre-threat and post-threat exposure of the male alone (top left panel) and with a female (second to fourth left panels) exposed to either an early threat (second middle left panel), a late threat (third left panel) or a late threat with a fixed abdomen (bottom left panel). The mean Δ*F*/*F*_0_ (%) comparing the pre-threat and post-threat time windows (*n* = 6 and 7) is also shown (right). Refer to the legend of Fig. 1 for details on graphics and statistics.

**Extended Data Fig. 6:**
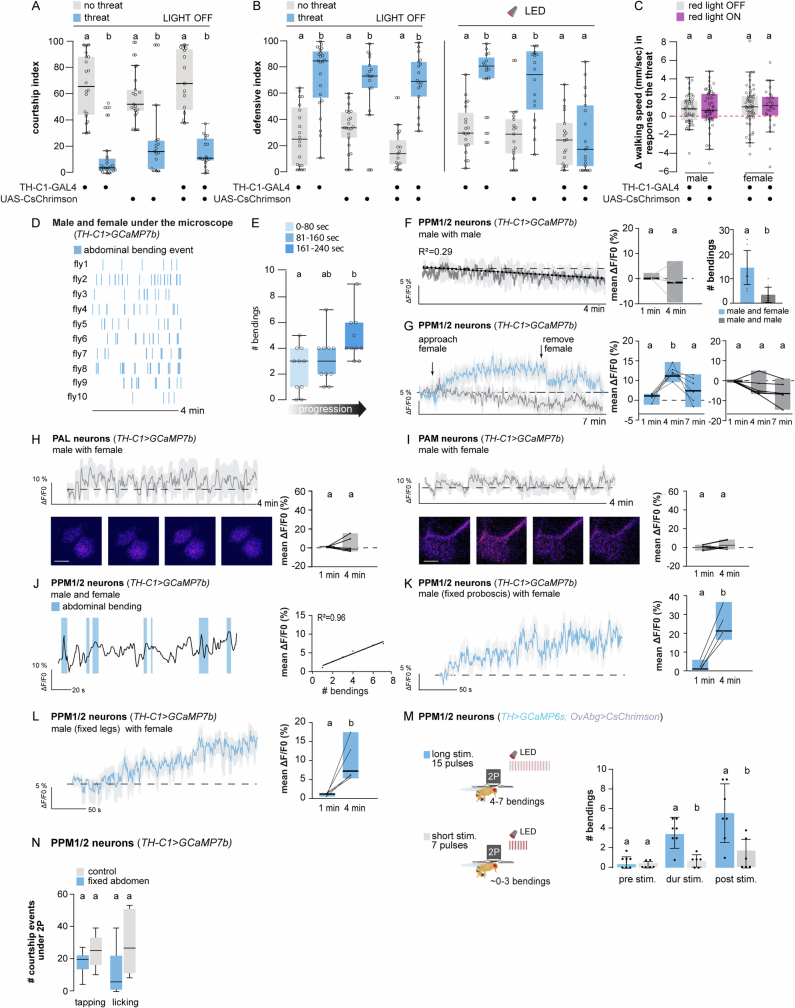
PPM1/2 dopamine neuron activity ramps up as courtship progresses. **A**, Courtship index of TH-C1-GAL4>CsChrimson without artificial activation, without (grey, n = 20,21,15) or with (blue, n = 23,17,18) threat. **B**, Defensive index of TH-C1>CsChrimson males without or with artificial activation, without (grey, n^light OFF^ = 20, 21, 15; n^light ON^ = 19, 17, 20) or with (blue, n^light OFF^ = 23, 17, 18; n^light ON^ = 16, 16, 21) threat. **C**, Change in walking speed before and after threat of TH-C1>CsChrimson males and females (light OFF (grey), n = 55-64, light ON (magenta), n = 60-28). **D**, Abdominal bending events of males TH-C1 > GCaMP7b paired with a female under the microscope. **E**, Abdominal bending events during the first, second, and third tiers of 4 min of male courting a female under the microscope (n = 10). **F**,**G**, ∆F/F0% of TH-C1^PPM1/2^ > GCaMP7b signal in males paired with a male **(F)** or female **(G)**. **F**, Mean ∆F/F0% during 1^st^ min (0-20 s) vs. at 4 min (220-240 s) (n = 5). Right: abdomen bending events (n = 10). **G**, ∆F/F0% in males paired with a female (blue) and control of adjacent ROI (grey). Right: mean ∆F/F0%  during 1^st^ (0-20), at 4 (220-240 s), and at 7 min (400-420 s) (n = 6). **H**,**I**, ∆F/F0% of TH-C1^PAL^ **(H)** and TH-C1^PAM^ > GCaMP7b **(I)** signal of males paired with a female. Right: comparing mean ∆F/F0% during 1^st^ to 4 min time windows (n = 6). Below: representative fluorescence heatmap (scale bar 6 µm). **J**, ∆F/F0% and alignment of abdominal bending events during recording of PPM1/2 neurons in TH-C1 > GCaMP7b males paired with a female. **K**,**L**, ∆F/F0% of TH-C1^PPM1/2^ > GCaMP7b signal in males with either a fixed proboscis **(K)** or fixed front legs **(L)** paired with a female. Right: mean ∆F/F0% during 1^st^ min and at 4 min (n = 6,5). **M**, LED stimulation protocol of Fig. 4e and number of evoked abdominal bendings of males THC1 > GCaMP6s; OvAbg>CsChrimson within the pre, during and post-stimulus windows (n = 5-6). **N**, Number of tapping or licking events of TH-C1 > GCaMP7b males paired with a female without or with fixed abdomen (n = 6-10). See legend to Extended Data Fig. 1 for details on graphics and statistics.

**Extended Data Fig. 7:**
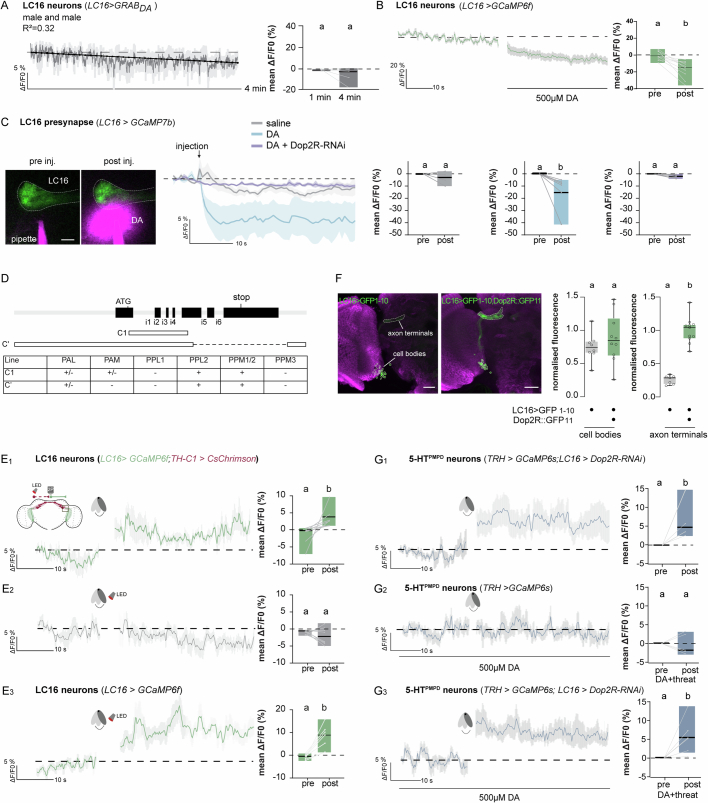
Copulation attempts drive dopaminergic inhibition of the visual threat pathway. **A**, ∆F/F0% of LC16 > GRAB_DA_ signal of males paired with a male fly. Right: mean ∆F/F0%  during 1^st^ min (0-20 s) and at 4 min (220-240 s) (n = 5). **B**, ∆F/F0% of LC16 > GCaMP6f signal of males pre and post application of 500 µM dopamine. Right: mean ∆F/F0% pre and post time windows (n = 10). **C**, ∆F/F0% of LC16 > GCaMP7b signal after focal injection of dopamine (blue), saline (grey), or dopamine in LC16 > GCaMP7b; Dop2R-RNAi males (magenta). Scale bar: 5 µm. Right: mean ∆F/F0% pre and post-injection. Saline (n = 5, 3 flies) vs. Dopamine (n = 6, 4 flies) vs. Dop2R-RNAi (n = 5, 3 flies). **D**, TH genomic region showing sequences upstream of Gal4 for lines C1 and C’. Exons are black boxes. ATG, stop, and i1-i6 mark translational start, stop, and intron sites. “+”, “+/−”, and “−” in the table indicate expression in most, a subset, or none of the neurons in the clusters, respectively. See^69^. Panel **D** is adapted from ref. ^69^, Elsevier. **E**_**1-3**_, ∆F/F0% of LC16>LexAop-GCaMP6f; TH-C1>CsChrimson signal pre and post: **E**_**1**_ threat, **E**_**2**_ threat with LED stimulation, or **E**_**3**_ threat and LED stimulation in control flies only expressing UAS-GCaMP6f in LC16 neurons. Right: Mean ∆F/F0% pre and post time windows (n = 8,8,7). **F**, Left: LC16 Split-GAL4 driving UAS-spGFP_1-10_ without (left) or with (right) tagging endogenous Dop2R::GFP_11_. Right: quantified GFP fluorescence in LC16 cell bodies (left) or axon terminals (right) with or without Dop2R::GFP_11_. anti-brp (nC82; magenta). (n = 8,10,7,11 brains). GFP fluorescence was normalised to the average fluorescence in the LC16 axon terminals in LC16 > GFP_1-10_,Dop2R::GFP_11_ flies. Scale bar: 25 μm. **G**_**1-3**_, ∆F/F0% TRH^PMPD^ > GCaMP6s pre and post threat **(G**_**1**_**)**, threat with 500 µM dopamine **(G**_**2**_**)**, and threat with 500 µM dopamine with additional knockdown of Dop2R in LC16 neurons **(G**_**3**_**)**. Right: mean ∆F/F0% of pre and post-time windows (n = 5,5,7). See legend to Extended Data Fig. 5 for details on graphics and statistics.

Having established that males become less responsive to threats as they advance in their courtship, we next explored how courtship progress is integrated within the neural circuitry that arbitrates between courtship and escape. Dopamine is linked with behaviour engagement and the perceived value and distance of a reward^22,26,46,47^. We therefore speculated that courtship progress might correlate with changes in dopamine neuron activity. To test this notion, we focused on dopamine neurons labelled by TH-C1-GAL4, some of which have been shown to modulate mating drive^19^ (Fig. 4a, Extended Data Fig. 7d and Supplementary Table 3). Activation of TH-C1 neurons during early courtship caused males to ignore threats and continue courting the female, although it did not affect courtship or visual threat detection per se (Fig. 4b and Extended Data Fig. 6a–c).

We thus asked whether TH-C1 dopamine activity might change with courtship progress. We developed a method for tracking neural activity as a male fly progresses through courtship under a two-photon microscope (Supplementary Video 6). When exposed to a virgin immobile female, tethered males displayed early and late courtship steps (for example, tapping, licking and abdomen bending). During the final 80 s of the experimental time window, males showed an increase in late courtship actions, such as abdomen bending (Extended Data Fig. 6d,e and Supplementary Video 6). GCaMP7b recordings revealed a gradual increase in calcium signal during courtship progression in a group of approximately seven dopamine neurons per hemisphere, named protocerebral posterior medial 1/2 (PPM1/2; Fig. 4c). This increase was absent when the focal male was paired with another male, which elicited minimal abdomen bending events (Extended Data Fig. 6f and Supplementary Table 2). The PPM1/2 calcium signal returned to baseline when the female was moved away (Extended Data Fig. 6g). In contrast to the ramping PPM1/2 signal, the GCaMP7b signal did not increase in adjacent cell bodies located in the same focal plane, or in TH-C1+PAL dopamine neurons (linked to mating drive^19^) or in TH-C1+PAM dopamine neurons (involved in courtship reward^48^) (Extended Data Fig. 6g–i and Supplementary Table 2). Together, these findings suggest that the calcium  ramping observed in PPM1/2 neurons is specific to courtship progression.

In line with our behavioural findings, ramping PPM1/2 calcium levels were correlated with abdomen bending (Fig. 4c, bottom right, and Extended Data Fig. 6j), suggesting a direct link between abdomen bending and dopamine activity. Indeed, PPM1/2 calcium signals increased during courtship even in males with immobilized probiscises or legs, indicating that PPM1/2 is not driven by licking or tapping the female (Extended Data Fig. 6k,l, Supplementary Table 2 and Supplementary Videos 8 and 9; note that males do not display wing vibrations in our setup, see Methods for details). Moreover, PPM1/2 activity was increased by stimulation of abdomen bending via OvAbg activation in solitary males (Fig. 4d, top). This effect was dose dependent, as dopamine ramping was observed after prolonged LED stimulation, which elicited 4–7 bending events, but not short LED stimulation, which elicited 0–3 bending events (Fig. 4d, middle and bottom panels, Extended Data Fig. 6m and Supplementary Table 2). Of note, males exhibited abdominal bending events even after the long LED stimulation ceased, suggesting that this ongoing behaviour may sustain elevated PPM1/2 calcium levels (Extended Data Fig. 6m). Our findings indicate that PPM1/2 dopamine ramping is primarily driven by abdomen bending during courtship progression.

The dopamine activity ramp could be driven by either sensory information triggered by the abdomen-bending action (proprioception) or the predictive signal in anticipation of this movement (efferent copy). To address this, we immobilized the abdomen of males, preventing males from bending it while preserving other courtship behaviours such as tapping and licking (Fig. 4e, Extended Data Fig. 6n and Supplementary Video 6). Following this manipulation, the GCaMP7b signal in PPM1/2 neurons did not show the expected ramping behaviour. Instead, PPM1/2 activity decreased significantly over time (Fig. 4e and Supplementary Table 2), suggesting that proprioceptive feedback from abdomen bending, rather than efferent copy from a command circuit, is required to ramp up dopamine activity in PPM1/2 neurons. Consistent with these findings, dopamine ramping triggered by optogenetic activation of OvAbg neurons only occurred when solitary males were physically able to bend their abdomen (Fig. 4d, top and bottom panel, and Supplementary Table 2). These findings predict that late-courtship abdomen bending suppresses LC16 visual threat responses. Indeed, in live-imaging experiments, we found that LC16 neurons responded to threats in solitary males and during early courtship (Fig. 4f, first and second panels) but not during late courtship (Fig. 4f, third panel). Of note, when abdomen bending was mechanically blocked, LC16 threat calcium responses during late courtship were restored (Fig. 4f, fourth panel and Supplementary Table 2). Together, our findings indicate that abdomen bending ramps up dopaminergic activity levels, which integrate proprioceptive feedback and ultimately induce a late-courtship state.

## Dopamine blocks visual threat detection

**Fig. 5:**
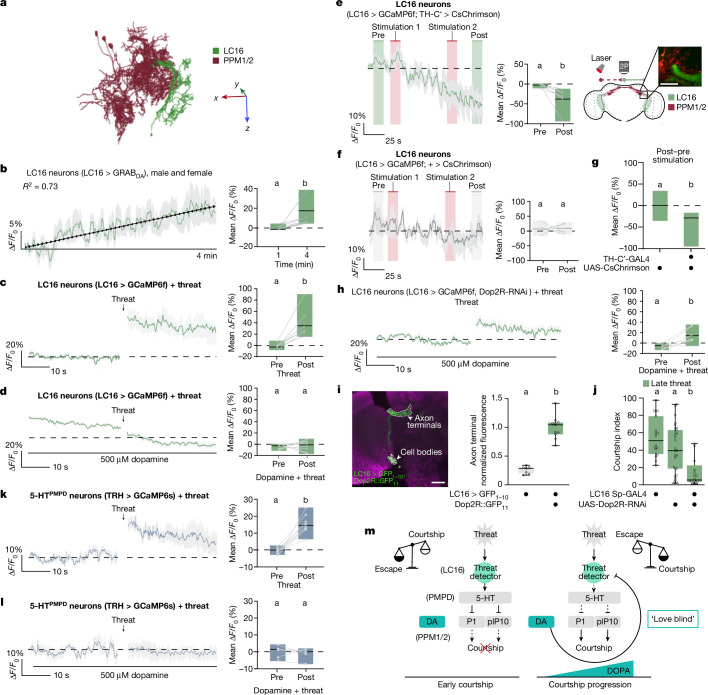
Ramping dopamine inhibits the visual threat pathway. **a**, Electron microscope reconstructions of female LC16 and PPM1/2. See Supplementary Information Fig. 5a for axis details. **b**, ∆*F*/*F*_0_ (%) of the LC16 > GRAB_DA_ signal of males paired with a female (left), and the mean ∆*F*/*F*_0_ (%) during the first minute (0–20 s) and at 4 min (220–240 s) (*n* = 7; right). **c**,**d**, ∆*F*/*F*_0_ (%) of the LC16 > GCaMP6f signal of males pre-threat and post-threat exposure, without (**c**) or with (**d**) 500 µM dopamine (left), and the mean ∆*F*/*F*_0_ (%) pre-threat and post-threat (*n* = 7 and 9; right). **e**,**f**, ∆*F*/*F*_0_ (%) of the LC16 > GCaMP6f signal of males with (**e**) or without (**f**) artificial activation of PPM1/2 neurons (left), the mean ∆*F*/*F*_0_ (%) pre-stimulation and post-stimulation (*n* = 8 and 6; middle), and a schematic and image showing the region of interest (right). Scale bar, 15 µm. **g**, Laser-induced ∆*F*/*F*_0_ (%; post–pre) signal of LC16 > GCaMP6f males with and without CsChrimson in PPM1/2 neurons. **h**, As in panel **d** but with Dop2R knocked down in LC16 neurons (left), and the mean ∆*F*/*F*_0_ (%) pre-stimulation and post-stimulation time windows (*n* = 7 and 9; right). **i**, UAS-spGFP_1–10_ driven by LC16 Sp-GAL4 combined with tagging endogenous Dop2R::GFP_11_ (left). Anti-brp (nC82) is in magenta. The fluorescence in axon terminals of LC16 > GFP_1–10_ neurons with or without Dop2R::GFP_11_ (normalized to the average for LC16 > GFP_1–10_, Dop2R::GFP_11_; *n* = 7 and 11) is also shown (right). Scale bar, 25 μm. **j**, LC16 > Dop2R-RNAi males respond to the late threat (*n* = 18, 29 and 20). **k**,**l**, ∆*F*/*F*_0_ (%) of the 5-HT^PMPD^ > GCaMP6s signal pre-threat and post-threat, without (**k**) or with (**l**) 500 µM dopamine (left), and the mean ∆*F*/*F*_0_ (%) pre-threat and post-threat time windows (*n* = 8 and 6; right). **m**, Working model. The solid and dashed lines indicate direct and indirect connections, respectively. DA, dopamine. Refer to the legend of Fig. 1 for details on graphics and statistics.

We next asked how increased dopamine levels might translate courtship progression into reduced threat responses. Using the female connectome^39^, we found that LC16 neurons receive direct input from PPM1/2 at their axon terminals but not from other dopaminergic clusters (Fig. 5a). Thus, we hypothesized that PPM1/2 neurons directly modulate the perception of visual threats.

To functionally test the PPM1/2–LC16 connection, we imaged dopamine release onto LC16 presynaptic terminals using a GPCR activation-based dopamine (GRAB_DA_) sensor^49^. We found a steady increase in GRAB_DA_ fluorescence in males paired with a female, indicating a gradual increase in dopamine release onto LC16 presynaptic terminals (Fig. 5b), consistent with the steady increase in PPM1/2 activity (Fig. 4c). This dopamine ramping was not observed in males paired with another male (Extended Data Fig. 7a and Supplementary Table 2). These findings suggest that the gradual release of dopamine onto LC16 may help to reduce responses of LC16 axonal terminals to visual threats during courtship progress.

To directly test whether dopamine shuts down visual threat responses in LC16 neurons, we recorded activity in LC16 presynaptic terminals during threat delivery while simultaneously administering dopamine. Not only did application of dopamine reduce LC16 baseline calcium activity (Extended Data Fig. 7b) but it also completely suppressed the threat-driven LC16 response (Fig. 5c,d and Supplementary Table 2). Moreover, focal injection of dopamine using a micropipette directly onto LC16 presynaptic terminals caused a robust decrease in LC16 calcium activity, suggesting axo-axonal inhibition of LC16 output by dopamine (Extended Data Fig. 7c). In addition, optogenetically stimulating PPM1/2 dopamine neurons gradually decreased the GCaMP6s signal in LC16 neurons (Fig. 5e–g and Supplementary Table 2) and — consistent with dopamine administration — reduced threat responses in LC16 (Extended Data Fig. 7e and Supplementary Table 2).

This dopamine-induced inhibition seems to act through dopamine D2-like receptors (Dop2R), as expressing Dop2R-RNAi in LC16 neurons partially rescued the threat response and prevented LC16 inhibition by focal dopamine injection (Fig. 5h and Extended Data Fig. 7c). We confirmed Dop2R expression in LC16 using reconstitution of split-GFP, in which we tagged endogenous Dop2R with spGFP_11_ and expressed cytoplasmic spGFP_1–10_ under LC16 split-GAL4 (Fig. 5i and Extended Data Fig. 7f). Reconstituted GFP signal was significantly higher than in split-GFP_1–10_-only controls in LC16 presynapses, but not in LC16 cell bodies (Fig. 5i and Extended Data Fig. 7f), indicating that Dop2R receptors are localized in the axon terminals, proximal to PPM1/2 neurons. Crucially, when flies expressing Dop2R-RNAi in LC16 neurons were tested in the behavioural assay, their behaviour shifted from courtship to defensive responses during late courtship stages (Fig. 5j). Our findings predict that dopaminergic inhibition of outgoing activity from LC16 neurons would prevent 5-HT^PMPD^ neuronal responses to the visual threat. Indeed, we found that although 5-HT^PMPD^ activity increased following threat delivery (Fig. 5k), this response was abolished by adding dopamine (Fig. 5l and Supplementary Table 2). Moreover, this dopamine-driven inhibition of 5-HT^PMPD^ threat responses was in turn blocked by knocking down Dop2R in LC16–GAL4 neurons (Extended Data Fig. 7g).

Together, our findings suggest that dopamine signalling from PPM1/2 acts through Dop2R to shut down LC16-mediated threat detection, allowing males to persist in courtship despite the presence of a threat.

## Discussion

Amorous adventures can lead us to pursue risky actions. This ‘love blind’ state emerges from a fundamental function of life: weighing up risks against opportunities^1^. Our study showcases this balancing act by demonstrating that imminent mating success disrupts threat perception in *Drosophila* males, rendering them ‘love blind’. We have shown that, upon detecting a visual threat, LC16 visual neurons trigger 5-HT-mediated inhibition of key courtship nodes (P1 and plP10), prompting flies to abort courtship (Fig. 2o). However, as courtship progresses (as reported by abdomen bending), the activity within PPM1/2 dopamine neurons gradually increases. PPM1/2 activity in turn suppresses LC16 activity, allowing flies to persist in courtship and ignore external threats when close to mating (Fig. 5m). Thus, risk–benefit arbitration is dynamically modulated by goal proximity and is under dopaminergic control.

Similar examples of risk–reward trade-offs abound in the animal kingdom. Animals become less risk-averse when the opportunity cost of dying (foregone future mating opportunities) is lower or the expected reproductive rewards are higher. For example, male mice become less afraid of predator odours after smelling oestrous female odours^2^, while male moths following pheromone plumes ignore ultrasound cues that simulate an approaching bat^3^. Our results provide a mechanistic framework for these classic ethological questions by revealing how risk versus reward trade-offs are calculated based on mating success.

Our study suggests that abdomen bending triggers a late-courtship state via proprioceptive feedback ramping up dopaminergic activity. PPM1/2 neurons do not necessarily respond to discrete abdominal-bending events with phasic, time-locked responses. Instead, the observed gradual ramping of PPM1/2 activity suggests that PPM1/2 neurons integrate proprioceptive inputs over time, leading to a gradual increase in tonic calcium levels. We propose that this gradual rise in calcium activity within dopaminergic neurons stems from the continued integration of both female sensory cues and proprioceptive feedback from abdominal bending. It will be interesting to test candidate biophysical mechanisms underlying this integration, such as neuromodulatory regulation of spontaneous activity and intrinsic excitability^50^, in future studies.

In addition to dopamine ramping suppressing threat detection, other parallel modulatory mechanisms might work together to prioritize courtship when copulation is imminent. Indeed, PPM1/2 activation prevents threat responses in courting males but not in solitary males, suggesting that PPM1/2 inhibits but does not entirely silence LC16 output, such that the reduced output can still drive escape behaviour in solitary males but cannot outcompete courtship drive. Parallel mechanisms might reduce serotonergic threat responses or reduce the sensitivity of central courtship nodes to serotonergic inhibition. For example, sexual arousal due to increased P1 activity gates the perception of female-related visual cues during courtship^15^; after courtship begins, P1 activity is thought to be sustained by recurrent activation, facilitated by dopamine released from SMPa neurons^16,19,28^ (although P1 activity does not ramp up in the way PPM1/2 neurons do^51^). Future experiments should test whether this recurrent activity is facilitated by the same PPM1/2 neurons that ramp up as courtship progresses.

Studies in mammals have reported dopamine ramping in diverse behavioural trials that lead to reward, including goal-directed navigation and multi-step tasks^24,25,46,47^. Such ramping release profiles have been proposed to supply the motivational drive required to sustain goal pursuit, as they scale with distance to reward^25,46,47,52–54^. Our study showed that in addition to its well-established role in encoding reward expectation^23,24^, dopamine ramping also serves as a gradual sensory filter system, which shuts down the visual threat pathway as courtship progresses. This mechanism would put animals in a ‘love blind’ state, allowing them to pursue their reproductive goal despite the danger.

Dopaminergic modulation of sensory signalling has been shown in many species. In lampreys and zebrafish, dopaminergic neurons modulate responses to visual features such as looming cues^55,56^. In rodents, dopamine influences subcortical responses to unexpected auditory stimuli^57^. In humans, antipsychotic drugs are thought to act on D2-like receptors^58^, the mammalian homologue of Dop2R, suggesting that D2-like receptors may have a common role in top-down regulation of sensory perception, whether in generating hallucinations or ignoring visual threats. Given the striking similarities in the cellular biology of dopamine neurons across species^26,57–60^, dopamine-mediated filter systems may be a general mechanism for blocking sensory cues that compete with more important goals.

## Methods

### Resource availability

Requests for additional information and reagents should be addressed to the lead author (C.R.). All data generated in this paper can be shared on request.

### Fly husbandry and strains

Flies were reared at 25 °C or 30 °C for RNAi experiments, with 40–50% humidity on a standard cornmeal-agar food in a 12-h light–dark cycle. Canton-S (CS) strain flies were used as wild type. Flies were sorted under CO_2_ anaesthesia within 6 h of emergence and housed in same-sex groups of 20, except for the males that were to be tested in the behavioural experiments, which were kept in groups of 4 per vial. Virgin females for the behavioural experiments were collected using the *hs-hid* conditional virginator transgenic line. L3 larvae were heat shocked at 37 °C for 1.5 h. Additional strains used and their sources^61–69^ are outlined in Supplementary Table 3.

### *Trans*-retinal food

*Trans*-retinal (R2500-100MG, CAS number: 116-31-4, Sigma-Aldrich) was stored at –20 °C as a 50 mM stock solution diluted in ethanol and wrapped in foil. To blend retinal homogeneously into the food, 60 μl of stock solution was directly pipetted into 6-ml vials of liquid cornmeal-yeast food except for the experiment in Fig. 3e in which OvAbg flies were not exposed to food supplemented with *trans*-retinal factor.

### Behaviour

#### Threat setup

Experiments were recorded at 27 frames per second using a Mako U-130B camera mounted with an infrared filter (BP735-40.5, Midopt). The visual threat was generated by repeatedly passing a 13 cm × 6 cm × 2 cm 3D-printed opaque oblong paddle through a blue-light beam (455 nm). This created an overhead shadow at periodic intervals of 0.3 Hz for 30 s. The paddle was set 5 cm above the courtship chambers (∅20 mm, 5 mm) at a 90^o^ angle on a servo motor controlled by a custom-built Arduino code, which controlled the movement parameters of the paddle (frequency set to 0.3 Hz and number of cycles set to 9). The mechanical threat was generated using a Sony XP500-X speaker playing a loud 3-Hz binaural beat (https://www.youtube.com/watch?v=Y-urmCRs61I&t=713s) causing surface vibrations. Courtship chambers were illuminated from the bottom using an infrared backlight.

#### Behavioural assays

Behavioural assays were conducted at 25 °C under continuous blue light between 09:00 and 13:00. Tested males were 5–7 days of age and transferred to fresh food vials 1 day before experiments. For males used in optogenetic assays, flies were transferred to food enriched with *trans*-retinal 3 days before the experiment. Vials containing retinal were wrapped in foil. Virgin females were decapitated and used within a maximum of 3 h to preserve chemical signature and motor reflexes during the experiment.

#### Action selection assay

The action selection assay presented a naive male coupled with a decapitated *hs-hid* virgin female with a choice between continuing to court the female or interrupting the ritual in response to the threat. The threat was delivered after consistent courtship of at least 7 s (early), 2 min (middle) or 4 min (late). Only males that started to court during the first 5 min of the trial and until threat delivery were considered in the analysis. All assays were manually analysed using the behavioural analysis software BORIS^70^, and the following parameters were quantified to measure the effect of the threat on male courtship behaviour.

The courtship index is defined by the percentage of time (in seconds) the male spends courting the female over the total time of the threat delivery (30 s). We considered that males initiated courtship by demonstrating full wing extension and a persistent courtship behaviour of at least 7 s towards the female. We considered courtship as the display of stereotyped courtship events that include tapping of the female with the forelegs of the male, singing (wing extension and vibration), licking (male proboscis extension) and attempts at copulation in which the male bends the abdomen towards the female and attempts to mount her. See Fig. 1a for a schematical representation of these behaviours.

The defensive index is defined by the percentage of time (in seconds) the male spends displaying defensive behaviours (that is, escaping and freezing) over the total time of the threat delivery (30 s).

As a control, the behaviour was assessed in the absence of the visual threat during the same time window according to the same criteria.

#### Optogenetic assay

Flies were tested in a transparent circular chamber (∅ 20 mm, H = 5 mm for courtship; and ∅ 24 mm, H = 3 mm for the locomotion assay) and illuminated from underneath with either 660-nm (red) or 515-nm (green) light in the absence or presence of the threat. Refer to Supplementary Table 4 for the optogenetic experimental conditions corresponding to each figure. The light was turned ON 1 s before the first threat passed.

#### Locomotion assay

Individual flies were introduced into a circular chamber (∅24 mm, H = 3 mm) and left to acclimatize for 3 min. After the acclimatation period, flies were subjected to the threat (9 cycles and frequency of 0.3 Hz). The walking speed of the flies (thresholded at values larger than 4 mm s^−1^ to be considered as ‘walking’) was assessed using the Ethovision XT17 software. The change in walking speed was calculated by subtracting the average walking speed of the 30 s after threat from the 30-s average before threat delivery.

#### Two-photon functional imaging

Tethered male flies (3–6 days of age) had their head capsules dissected in a sugar-free HL3-like saline-filled imaging chamber with a central hole (for details on fly dissection, see ref. ^71^). Flies were then placed under a multiphoton microscope (Femto2D-Resonant by Femtonics), and expressed either the calcium indicator GCaMP or GRAB_DA_ in different sets of neurons (see Supplementary Table 3 for details on genotypes). Fluorescence was generated by a Ti-Sapphire laser centred on 920 nm (Chameleon Ultra II, Coherent). Images with a pixel size of 0.3 × 0.3 μm were acquired with a ×20, 1.0 NA water-immersion objective, controlled by the MESc v3.5 software (Femtonics). Fast recordings were taken at a speed of 30 Hz with a resonant scan head using MESc software (Femtonics). Analysis was performed using NOSA software v1.1.16 (neuro-optical signal analysis)^72^ and a customized R script or Graphpad Prism, Regions of interest (ROIs) were manually drawn for analysis. Data were converted into tiff files and processed using a Savitzky–Golay filter or moving average of 2 s when brain movement was strong (Figs. 4c and 5b). No baseline/photobleaching correction was applied to any of the imaging data. The final time resolution was 6 fps (Femtonics microscope data) or 2 fps (Optogenetic data from Nikon microscope). Mean intensity values were calculated as Δ*F*/*F*_0_ (in %), whereas *F*_0_ was defined as the mean *F* from baseline activity (first 30 s in Figs. 1h,i, 2i,j,m,n, 4e and 5c,d,h,k,l and Extended Data Figs. 3j, 4h, 7b,e; the first 20 s in Figs. 4c,f, and 5b and Extended Data Figs. 6f–i,k,l and 7a; the first 15 s in Fig. 5e,f and Extended Data Fig. 7e,g; and the first 2 s in Fig. 2e and Extended Data Fig. 3b).

#### Threat delivery under the two-photon microscope

The threat was delivered as previously described (see the ‘Threat setup’ section). The paddle and light source were placed below the microscope and inclined towards the chamber in a way that the passing shadow reached the tethered fly’s eye. Calcium signals in LC16 axons and PMPD neurons were recorded for 30 s before and 60 s immediately after the threat exposure (calculation windows in Figs. 1h,i and 5c,d,h,k,l: last 10 s before and first 10 s after; Fig. 4f and Extended Data Fig. 7e,g: last 15 s before and 30 s after). As LC16 neurons respond to laser onset, the first 2 s of each recording were excluded from the analysis. Conditions under the microscope were set to more than 20 °C and 40% humidity.

#### Application of serotonin or dopamine

100 µl of serotonin (H9523, Sigma-Aldrich) or dopamine (H8502, Sigma-Aldrich) diluted in sugar-free HL3 solution was applied directly onto the *Drosophila* brain through the open head capsule. The final concentration was 100 µM for serotonin and 500 µM for dopamine. Calcium signals were recorded 50 s before and 100 s immediately after application (first 30 s of pre-application and last 30 s of post-application were taken for quantification).

#### Courtship progression under the microscope

For examining courtship progression, 5–8-day-old virgin male flies were used. Flies were tethered and dissected as previously described, leaving legs and proboscis freely moveable (or fixed depending on the experiment indicated for each figure). Note that the fixation position of the male onto the imaging chamber does not allow for wing extension. Agitated males that did not stop moving for 10 s during the first 5 min under the microscope were discarded. Immediately upon recording initiation, a decapitated 3–5-day-old virgin female tethered onto a moveable arm controlled by a micromanipulator was presented to the male with her abdomen oriented towards the head of the male fly. Following male contact with the female, calcium or GRAB_DA_ signals were recorded for a total duration of 4 min, while the fly behaviour was simultaneously observed using a video camera (Thorlabs C1285R12M and SM1D12D iris diaphragm) recording at 7 fps. The first 20 s and last 20 s were taken for quantification (except Extended Data Fig. 6g: 1–20 s, 240–260 s and 400–420 s). Abdomen bending was manually analysed frame by frame. As tethered flies show typical behaviour that includes moving the abdomen back and forth, only full-bending events (the tip of the abdomen bending underneath the thorax) that lasted longer than 1 s or 6 frames were considered as part of courtship behaviour.

#### Optogenetic experiments during in vivo calcium imaging

Experiments were conducted using a Nikon A1R+ multiphoton microscope with a galvo scanner at a speed of 2 Hz. We used the two-photon 1,040-nm red laser of the microscope to activate CsChrimson while simultaneously recording the calcium activity within the ROI (see the details for the conditions in the main text figure legends and Supplementary Table 4). To activate OvAbg neurons, experiments were carried out using a Femtonics microscope with the same imaging parameters mentioned previously. A 590-nm LED positioned below and towards the tethered fly was used for optogenetic activation of CsChrimson (15 or 7 repetitions of 1-s LED-on and 1-s LED-off intervals) while recording simultaneously. To activate PPM1/2 neurons during threat delivery, 15 repetitions of red light were used overlapping the 30 s of threat exposure under the microscope. LED stimulation artefacts were removed for clarity. As the acquisition was carried out continuously, the post-sequence shown in the graph displays the fluorescence intensity immediately after the LED stopped (Fig. 4d).

#### Focal dopamine injection

Fly preparation and imaging were conducted as described previously^40^ using a Nikon A1R+ multiphoton microscope. The sugar-free HL3-like saline was added with 30 units of Papain (Roche) and applied to the head capsule for 10 min to digest the glial sheath of the brain and facilitate removal. Flies were subjected to local dopamine (10 mM diluted in saline) or saline injection via a micropipette (saline used for injection contained no CaCl_2_ or MgCl_2_). The injection solution was labelled with Texas Red (Invitrogen by Thermo Fisher Scientific, dextran, 10,000 MW) to visualize the pipette and the localization of the injections. Multiple (2–5) injections were given per experiment and averaged, resulting in a single average trace per experiment. Fluorescence traces were extracted using FIJI (ImageJ). *F*_0_ for the Δ*F*/*F* calculations was the average baseline fluorescence of the 10 frames immediately preceding the injection. Calculation windows for mean Δ*F*/*F*_0_ % was 10 s pre and last 10 s post. ROIs were selected manually.

#### Immunohistochemistry

Three-to-five-day-old male fly brains were dissected in ice-cold PBS and fixed in 4% paraformaldehyde solution at room temperature for 20 min. Fixed brains were then washed four times in PBST (0.5%) for 30 min and blocked with normal goat serum (5%) for 30–60 min. The brains were then incubated with primary antibodies (anti-GFP chicken, 1:1,000 or 1:2,000, 13970, Abcam; anti-dsRed rabbit, 1:250, 632496, Takara; and nC82 anti-Brp, 1:50, DSHB) for 2–3 days at 4 °C. After four 20-min washes in PBST, the brains were incubated overnight with secondary antibodies (Alexa Fluor 488 goat anti-chicken IgG, 1:1,000 (A28175) or 1:2,000 (A32931), Thermo Fisher Scientific); Alexa Fluor 546 goat anti-mouse, 1:2000, A11018, Thermo Fisher; and Alexa Fluor 546 goat anti-rabbit, 1:2,000, A11071, Thermo Fisher). After four 20-min washes in PBST, brains were mounted in Vectashield on a glass slide before scanning with a Leica SP8 confocal microscope, a Nikon A1 confocal microscope or a Zeiss LSM900 with AiryScan2 module.

#### Split-GFP immunohistochemistry

Three-to-seven-day-old male fly brains were dissected in room temperature PBS and fixed in 4% paraformaldehyde solution at room temperature for 20 min. Fixed brains were then washed in PBST (0.3%) three times for 20 min each and blocked with normal goat serum (5%) for 30 min. The brains were then incubated with anti-Brp (nC82, 1:50, DSHB) with 5% goat serum for 2 days at 4 °C. No anti-GFP antibody was used. After three 20-min washes in PBST, the brains were incubated with Alexa Fluor 546 goat anti-mouse (1:2,000, A11018, Thermo Fisher) for 2 days at 4 °C. After four 20-min washes in PBST, brains were mounted in Vectashield on a glass slide before scanning with a Nikon A1 confocal microscope.

Reconstituted split-GFP signal was quantified using ImageJ. The GFP signal was taken as the average pixel intensity within manually drawn volumes (freehand ROIs in multiple *z*-slices) around the LC16 axon terminals and cell bodies. The background fluorescence (from an ROI in a proximal brain region outside the LC16 neuron) was subtracted from the GFP signal. Statistical significance was evaluated by *t*-tests and two-way ANOVA in GraphPad Prism 9.

#### Connectomics search

We used the neuprint (hemibrain v1.2.1 dataset)^39^ platform to search for candidate neurons and subsequent connectivity (https://neuprint.janelia.org/).

- Predicted link between LC16 and pC1a: Query Selection > General > Shortest paths > neuron A = LC16 # 1256830582 > Neuron B = pC1a # 359744514, Minimum weight = 3.
- 3D visualization of 5-HT^PMPD01^ and pC1 neurons: ‘dataset’:‘hemibrain:v1.2.1’,‘bodies’[‘297230760’,‘\n297908801’,‘\n359744514’,‘\n5813046951’,‘\n267214250’,‘\n267214250’,‘\n392821837’,‘\n359744514’,‘\n5813046951’,‘\n514850616’].
- 3D visualization of LC16 neurons and PPM1/2 neurons: ‘dataset’:‘hemibrain:v1.2.1’,‘bodies’[‘1350945956’,‘1288897930’,‘1319927345’,‘1319587380’,‘1319579391’,‘1254037524’,‘1288893503’,‘1289238972’,‘1319586861’,‘1319919918’,‘1412989088’,‘950229431’,‘792040520’,‘5813054384’].

#### Statistics and reproducibility

See Supplementary Tables 1 and 2 for details on statistics. All statistical tests were performed using R v2023.03.1 + 446 or GraphPad Prism 9. Each behavioural experiment was repeated at least three times over a minimum of 3 days. Individuals were tested only once. The sample size for the behavioural experiments always represents biologically independent animals. Behavioural indexes and calcium imaging quantification are displayed as boxplots. Boxes represent the lower (25th) and upper (75th) interquartile, respectively, and the horizontal line represents the median. Each dot on the plot represents a single fly. Courtship progression behavioural data and locomotion data do not follow a normal distribution, thus non-parametric Mann–Whitney or Kruskal–Wallis tests, followed by a Conover–Iman multiple pairwise comparisons post-hoc test, have been applied on raw data (*P* = 0.05, with a Bonferroni correction) for one factor experiments. To test the interaction between the genetic manipulations and the treatments, we applied two-way ANOVA. Significant differences are indicated by different letters at the level of *P* < 0.05. We used a one-sample Wilcoxon signed-rank test (*μ* = 0) to assess whether the speed change (∆) in Extended Data Fig. 5e significantly deviated from 0. We indicated significance using an asterisk at the level of *P* < 0.05.

Calcium imaging traces over time are represented as the mean ∆*F*/*F*_0_ (%; solid lines) with s.e.m. (shaded area). Quantification plots are shown as minimum/maximum plots and the median as the horizontal line. After verification of normality, a paired *t*-test or paired Wilcoxon signed-rank test was applied on mean ∆*F*/*F*_0_ (%) data from individual flies on specific time windows indicated in the figures and/or in the Methods. Significant differences are indicated by different letters (*P* < 0.05). For inter-group comparisons, mean pre values were subsracted from mean post values and differences between genotypes and treatments were tested using one-way ANOVA, Kruskal-Wallis, t-test or Mann-Withney test as approriate. Experimenters were not blinded to the conditions of the experiments during data collection. Genotypes used for one experiment were tested simultaneously and in random order as well as random times during the day to avoid any influence of circadian timepoints and order of the experimental trials. We repeated all statistical tests excluding data points that were identified as outliers using the ROUT method in Prism with *Q* = 0.5%, and always obtained the same results, so we did not exclude outlier data points. Expression pattern of TH-C1-GAL4 and split-GAL4 lines, including LC16, P1, TRHR^23E12^ and plP10, were all imaged in *n* = 4 flies and were reliable across samples.

#### Randomization and blinding

Animals were never pre-assigned to a treatment or control group before the experiments. Behavioural and imaging experiments were performed in conjunction with their respective control cohorts. Randomization of animals was not implemented in this design.

### Reporting summary

Further information on research design is available in the Nature Portfolio Reporting Summary linked to this article.

## Online content

Any methods, additional references, Nature Portfolio reporting summaries, source data, extended data, supplementary information, acknowledgements, peer review information; details of author contributions and competing interests; and statements of data and code availability are available at 10.1038/s41586-024-07890-3.

## Supplementary information

Supplementary Information This file contains table of contents for figures 2, 4 & 5; Supplementary Tables 1–4 and Supplementary Videos 1–9

Reporting Summary

Supplementary Table 1 Statistics for behavioural, live calcium imaging and anatomical data (main and extended figures)

Supplementary Table 2 live calcium imaging inter-group comparisons

Supplementary Table 3 list of strains and genotypes

Supplementary Table 4 list of the optogenetic conditions per genotype and figure

Supplementary Video 1 Wild-type CS male displaying early courtship steps toward an immobile female.

Supplementary Video 2 Wild-type CS male displaying late courtship steps toward an immobile female.

Supplementary Video 3 Wild-type CS male displaying freezing behaviour in response to an early threat, delivered 7 s after courtship initiation

Supplementary Video 4 Wild-type CS male running away in response to an early threat, delivered 7 s after courtship initiation

Supplementary Video 5 Wild-type CS male pursuing courtship and displaying abdominal bending in the presence of a late threat, delivered 4 min after courtship initiation

Supplementary Video 6 Tethered wild-type male CS displaying abdominal bending toward a female under the two-photon microscope

Supplementary Video 7 Tethered TH-C1> GCaMP7bmale with fixed abdomen paired with a female under the two-photon microscope

Supplementary Video 8 Tethered TH-C1>GCaMP7b male with a fixed proboscis paired with a female under a two-photon microscope.

Supplementary Video 9 Tethered TH-C1>GCaMP7b male with fixed front legs paired with a female under a two-photon microscope.

## Data Availability

Source data are available at https://github.com/lczl64/Cazale-Debat-Scheunemann-et-al.

## Code availability

Codes are available at https://github.com/lczl64/Cazale-Debat-Scheunemann-et-al and https://github.com/jthueringer/NosaAnalysis.
