# Supplementary material for: Mating proximity blinds threat perception
Source: Nature. 2024 Aug 28;634(8034):635–43. doi: 10.1038/s41586-024-07890-3 (PMC11485238; doi:10.1038/s41586-024-07890-3)
Supplement: Supplementary file 1 — This file contains table of contents for figures 2, 4 & 5; Supplementary Tables 1–4 and Supplementary Videos 1–9 [file 41586_2024_7890_MOESM1_ESM.docx]

| **SUPPLEMENTARY INFORMATION GUIDE** |
| --- |

**Mating proximity blinds threat perception.**

Laurie Cazalé-Debat^1,2,10^, Lisa Scheunemann^3,4,10^, Megan Day^1,2^, Tania Fernandez-d.V. Alquicira^4^, Anna Dimtsi^1,2,7^, Youchong Zhang^1,2,8^, Lauren A. Blackburn^1,2,9^, Charles Ballardini^1,2,^ Katie Greenin-Whitehead^5,6,^ Eric Reynolds^4^, Andrew C. Lin^5,6,11^, David Owald^4,11^ & Carolina Rezaval ^1,2^

Table of Contents

[Supplementary Information Figures’ Legends. 2](#_Toc169533340)

[Figure 2 2](#_Toc169533341)

[Figure 4: 2](#_Toc169533342)

[Figure 5 2](#_Toc169533343)

[SUPPLEMENTARY TABLES: 2](#_Toc169533344)

[SUPPLEMENTARY VIDEOS 2](#_Toc169533345)

# Supplementary Information Figures’ Legends.

## Figure 2

**(C)** x,y,z arrows represent the anterior/posterior, medial/lateral and dorsal/ventral axis, respectively

**(D)** Bottom panel: schematic representation of the different anterior (black, ‘a’) and posterior (grey, ‘p’) serotonin clusters in the adult central brain. **1:** **PMPD** (posterior medial dorsal protocerebrum), **2:** **ADMP** (anterior dorsomedial protocerebrum), 3: **ALP** (anterior lateral protocerebrum), **4:** **PMPM** (posterior medial protocerebrum medial), 5: **PLP** (posterior lateral protocerebrum), **6:** **AMP** (anterior medial protocerebrum), **7:** **LP** (lateral protocerebrum), **8:** **SEL** (lateral subesophageal ganglion), **9:** **SEM** (medial subesophageal ganglion), **10: PMPV** (posterior medial protocerebrum ventral).

## Figure 4:

**(A)** Bottom panel: schematic representation of TH-C1-GAL4 expression pattern (blue) and the different anterior (grey) and posterior (black) dopamine clusters in the adult central brain. **1:** **PAL** (protocerebral anterior lateral), **2: PAM** (protocerebral anterior medial), **3: PPM1** (protocerebral posterior medial 1), **4: PPM2** (protocerebral posterior medial 2), 5**: PPL1** (posterior protocerebrum lateral 1), **6: PPL2c** (posterior protocerebrum lateral 2c), **7: PPM3** (protocerebral posterior medial 3), **8: PPL2ab** (posterior protocerebrum lateral 2ab), **9: T1** (thoracic 1), **10: SB** (subesophageal zone).

## Figure 5

**(A)** x,y,z arrows represent the anterior/posterior, medial/lateral and dorsal/ventral axis, respectively

# **SUPPLEMENTARY TABLES:**

Supplementary Table 1: Statistics for behavioural, live calcium imaging and anatomical data (main and extended figures).

Supplementary Table 2: live calcium imaging inter-group comparisons

Supplementary Table 3: list of strains and genotypes

Supplementary Table 4: list of the optogenetic conditions per genotype and figure.

# **SUPPLEMENTARY VIDEOS**

Supplementary Video 1: Wild-type CS male displaying early courtship steps toward an immobile female.

Supplementary Video 2: Wild-type CS male displaying late courtship steps toward an immobile female.

Supplementary Video 3: Wild-type CS male displaying freezing behaviour in response to an early threat, delivered 7 s after courtship initiation.

Supplementary Video 4: Wild-type CS male running away in response to an early threat, delivered 7 s after courtship initiation.

Supplementary Video 5: Wild-type CS male pursuing courtship and displaying abdominal bending in the presence of a late threat, delivered 4 min after courtship initiation.

Supplementary Video 6: Tethered wild-type male CS displaying abdominal bending toward a female under the two-photon microscope.

Supplementary Video 7: Tethered TH-C1> GCaMP7bmale with fixed abdomen paired with a female under the two-photon microscope.

Supplementary Video 8-9: Tethered TH-C1>GCaMP7b males with either a fixed proboscis or fixed front legs, respectively, paired with a female under the two-photon microscope.
