# Supplementary material for: Mating proximity blinds threat perception
Source: Nature. 2024 Aug 28;634(8034):635–43. doi: 10.1038/s41586-024-07890-3 (PMC11485238; doi:10.1038/s41586-024-07890-3)
Supplement: Supplementary file 2 — Reporting Summary [file 41586_2024_7890_MOESM2_ESM.pdf]

nature portfolio

Corresponding author(s): Carolina Rezaval

Last updated by author(s): 16 June 2024

Reporting Summary

Nature Portfolio wishes to improve the reproducibility of the work that we publish. This form provides structure for consistency and transparency in reporting. For further information on Nature Portfolio policies, see our Editorial Policies and the Editorial Policy Checklist.

Please do not complete any field with "not applicable" or n/a. Refer to the help text for what text to use if an item is not relevant to your study. For final submission: please carefully check your responses for accuracy; you will not be able to make changes later.

Statistics

For all statistical analyses, confirm that the following items are present in the figure legend, table legend, main text, or Methods section.

n/a	Confirmed	
☐	☒	The exact sample size (n) for each experimental group/condition, given as a discrete number and unit of measurement
☐	☒	A statement on whether measurements were taken from distinct samples or whether the same sample was measured repeatedly
☐	☒	The statistical test(s) used AND whether they are one- or two-sided<br>*Only common tests should be described solely by name; describe more complex techniques in the Methods section.*
☐	☐	A description of all covariates tested
☐	☒	A description of any assumptions or corrections, such as tests of normality and adjustment for multiple comparisons
☐	☒	A full description of the statistical parameters including central tendency (e.g. means) or other basic estimates (e.g. regression coefficient) AND variation (e.g. standard deviation) or associated estimates of uncertainty (e.g. confidence intervals)
☐	☒	For null hypothesis testing, the test statistic (e.g. F, t, r) with confidence intervals, effect sizes, degrees of freedom and P value noted<br>*Give P values as exact values whenever suitable.*
☒	☐	For Bayesian analysis, information on the choice of priors and Markov chain Monte Carlo settings
☒	☐	For hierarchical and complex designs, identification of the appropriate level for tests and full reporting of outcomes
☒	☐	Estimates of effect sizes (e.g. Cohen's d, Pearson's r), indicating how they were calculated

Our web collection on statistics for biologists contains articles on many of the points above.

Software and code

Policy information about availability of computer code

Data collection	Visual stimuli were generated using a 3D printed paddle run by a custom Arduino (version 1.8.15) script (stl and code files are available in https://github.com/lczl64/Cazale-Debat-Scheunemann-et-al). Locomotion data were acquired using Ethovision X17. Courtship behaviour was assessed using the Behavioral Observation Research Interactive Software (BORIS v. 7.13.9).Imunofluorescent images were acquired using a Leica SP8 confocal microscope and a Zeiss LSM900 with AiryScan2 module. Two-photon calcium imaging was performed using a Femto2D-Resonant by Femtonics Ltd., Hungary. Images with a pixel size of 0.3 × 0.3 μm were acquired with a 20×, 1.0 NA water- immersion objective, controlled by MESc v3.5 software (Femtonics Ltd., Hungary). Fast recordings were taken at a speed of 30 Hz with a resonant scan head using the mesc software (Femtonics). In-vivo imaging involving optogenetic treatment were conducted using a Nikon A1R+ multiphoton microscope with a galvo scanner with a speed of 2HZ. The code is available at https://github.com/lczl64/Cazale-Debat-Scheunemann-et-al.
Data analysis	We used R(2023.03.1+446, with embedded statistical and conover.test packages, graphics were plotted using the Beeswarm package), GraphPad Prism 9. Immunofluorescent images were visualized using Fiji and Imaris for 3D image processing. Fiji (ImageJ) version 2.0.0-rc71/1.52p and Imaris version 9.1 were used for image analysis. For calcium imaging, analysis were performed using Nosa software (v1.1.16) and a customized R script. ROIs were manually drawn for analysis. Data was converted into tiff files and processed using a Savitzky-Golay filter or moving average of 2 sec when movement was strong (Figure 5E). Code files are available in the github repository https://github.com/lczl64/Cazale-Debat-Scheunemann-et-al and https://github.com/jthueringer/NosaAnalysis for imaging analysis

For manuscripts utilizing custom algorithms or software that are central to the research but not yet described in published literature, software must be made available to editors and reviewers. We strongly encourage code deposition in a community repository (e.g. GitHub). See the Nature Portfolio guidelines for submitting code & software for further information.

Data

Policy information about availability of data

All manuscripts must include a data availability statement. This statement should provide the following information, where applicable:

- Accession codes, unique identifiers, or web links for publicly available datasets
- A description of any restrictions on data availability
- For clinical datasets or third party data, please ensure that the statement adheres to our policy

Codes and source data are available at https://github.com/lczl64/Cazale-Debat-Scheunemann-et-al

Research involving human participants, their data, or biological material

Policy information about studies with human participants or human data. See also policy information about sex, gender (identity/presentation), and sexual orientation and race, ethnicity and racism.

Reporting on sex and gender	N/A
Reporting on race, ethnicity, or other socially relevant groupings	N/A
Population characteristics	N/A
Recruitment	N/A
Ethics oversight	N/A

Note that full information on the approval of the study protocol must also be provided in the manuscript.

Field-specific reporting

Please select the one below that is the best fit for your research. If you are not sure, read the appropriate sections before making your selection.

☒ Life sciences ☐ Behavioural & social sciences ☐ Ecological, evolutionary & environmental sciences

For a reference copy of the document with all sections, see nature.com/documents/nr-reporting-summary-flat.pdf

Life sciences study design

All studies must disclose on these points even when the disclosure is negative.

Sample size	Sample sizes were not predetermined, but considered appropriate based on the field of research and recent papers with similar behavioural and tethered imaging preparations (Shen et al.2023; Cury & Axel, 2023; Hindmarsh Sten et al.2021)
Data exclusions	For courtship behaviour experiments, only males that started to court during the first five minutes of the trial and until threat delivery were considered in the analysis. No data were excluded in the analysis of the flies fitting the aforesaid criteria. For the locomotion assay unless in the event of a tracking acquisition error or data corruption (e.g. dropped frames, loss of the centroid), no data were excluded. For in vivo calcium imaging of LC16 neurons, the first 2 seconds of the recordings were cut off for analysis as they responded to laser onset. Concerning the courtship progression experiments under the microscope, agitated males that did not stop moving for 5min under the microscope were discarded. In addition, as tethered flies show typical behavior that includes moving the abdomen back and forth, only full bending events (abdomen bending underneath the thorax) that lasted longer than 6 frames were taken into account as part of courtship behavior. Acquisition in which brain movements to an extend in z that the ROI moved out of the focal plane were excluded from the dataset (only applicable to courtship progression since proboscis movement can lead to brain displacement, and to focal dopamine injection since dopamine puffs from the micropipette can lead to movement of the surrounding tissue).
Replication	Experiments were conducted and consistently reproduced across a diverse range of time scales, spanning from a minimum of three days to months or even years. Each experimental run involved no fewer than 5 individual flies for imaging and 7 for behaviour, and they were systematically evaluated alongside their corresponding genetic and treatment control groups.
Randomization	Animal were never pre-assigned to a treatment or control group prior to the experiments. Behavioral and imaging experiments were performed in conjunction with their respective control cohorts. For some of the imaging experiments, controls were tested within the same subjects, where each individual fly underwent followed periods of "sham" and treatment exposures. The randomization of animals was not implemented in this design.
Blinding	Blinding has not been implemented in this study. The assessment of behavior was assessed upon objective, dependable, and replicable measurable criteria.

Reporting for specific materials, systems and methods

We require information from authors about some types of materials, experimental systems and methods used in many studies. Here, indicate whether each material, system or method listed is relevant to your study. If you are not sure if a list item applies to your research, read the appropriate section before selecting a response.

Materials & experimental systems

n/a	Involved in the study
☐	☒ Antibodies
☒	☐ Eukaryotic cell lines
☒	☐ Palaeontology and archaeology
☐	☒ Animals and other organisms
☒	☐ Clinical data
☒	☐ Dual use research of concern
☒	☐ Plants

Methods

n/a	Involved in the study
☒	☐ ChIP-seq
☒	☐ Flow cytometry
☒	☐ MRI-based neuroimaging

Antibodies

Antibodies used	Primary anti body used were anti-GFP chicken Abcam 1:1000 (Cat#13970) and Alexa Fluor 488 goat anti-chicken IgG ThermoFisher Scientific 1:1000 (Cat#A28175). Secondary antibodies Alexa Fluor 488 goat anti-chicken IgG ThermoFisher Scientific 1:1000 (Cat#A28175 or A32931 1:2000), Alexa Fluor 546 goat anti-mouse, 1:2000, (Cat#A11018, ThermoFisher), Alexa Fluor 546 goat anti-rabbit, 1:2000 (ThermoFisher Cat#A11071).
Validation	The antibodies used are commercially sourced and have undergone multiple validation for their utility in the Drosophila immunohistochemistry community (https://www.thermofisher.com/antibody/primary/query/drosophila?ICID=srch-uc-antibodies, https://www.janelia.org/project-team/flylight/protocols).

Animals and other research organisms

Policy information about studies involving animals; ARRIVE guidelines recommended for reporting animal research, and Sex and Gender in Research

Laboratory animals	Drosophila melanogaster aged 3-8 days. Detailed descriptions of breeding, maintenance and genotypes are provided in extended methods and supplementary table 1 (including references to origins and stock numbers).
Wild animals	No wild animals were used in this study.
Reporting on sex	Only male flies were considered in the behavioural and imaging experiments of this study as it focuses on a male specific behaviour. The connectome dataset mentioned in this study were obtained from a female fly brain (Scheffer et al. 2020).
Field-collected samples	No samples were collected from the field in this study.
Ethics oversight	No ethical approval is necessary for research involving Drosophila melanogaster within the United Kingdom / Europe, where this study was conducted.

Note that full information on the approval of the study protocol must also be provided in the manuscript.

Plants

Seed stocks	N/A
Novel plant genotypes	N/A
Authentication	N/A

This checklist template is licensed under a Creative Commons Attribution 4.0 International License, which permits use, sharing, adaptation, distribution and reproduction in any medium or format, as long as you give appropriate credit to the original author(s) and the source, provide a link to the Creative Commons license, and indicate if changes were made. The images or other third party material in this article are included in the article's Creative Commons license, unless indicated otherwise in a credit line to the material. If material is not included in the article's Creative Commons license and your intended use is not permitted by statutory regulation or exceeds the permitted use, you will need to obtain permission directly from the copyright holder. To view a copy of this license, visit http://creativecommons.org/licenses/by/4.0/
